# Mussel-inspired multifunctional surface through promoting osteogenesis and inhibiting osteoclastogenesis to facilitate bone regeneration

**DOI:** 10.1038/s41536-022-00224-9

**Published:** 2022-05-13

**Authors:** Minhao Wu, Yufeng Zhang, Ping Wu, Feixiang Chen, Zhiqiang Yang, Sheng Zhang, Lingfei Xiao, Lin Cai, Chong Zhang, Yun Chen, Zhouming Deng

**Affiliations:** 1 Department of Spine Surgery and Musculoskeletal Tumor, Zhongnan Hospital of Wuhan University, 168 Donghu Street, Wuchang District, Wuhan, 430071 Hubei China; 2 College of Life Science and Technology Huazhong University of Science and Technology, Wuhan, 430074 China; 3 Department of Biomedical Engineering and Hubei Province Key Laboratory of Allergy and Immune Related Diseases, School of Basic Medical Sciences, Wuhan University, Wuhan, 430071 China

**Keywords:** Biomaterials - cells, Tissue engineering, Drug delivery

## Abstract

Osteogenesis and osteoclastogenesis are closely associated during the bone regeneration process. The development of multifunctional bone repair scaffolds with dual therapeutic actions (pro-osteogenesis and anti-osteoclastogenesis) is still a challenging task for bone tissue engineering applications. Herein, through a facile surface coating process, mussel-inspired polydopamine (PDA) is adhered to the surface of a biocompatible porous scaffold followed by the immobilization of a small-molecule activator (LYN-1604 (LYN)) and the subsequent in situ coprecipitation of hydroxyapatite (HA) nanocrystals. PDA, acting as an intermediate bridge, can provide strong LYN immobilization and biomineralization ability, while LYN targets osteoclast precursor cells to inhibit osteoclastic differentiation and functional activity, which endows LYN/HA-coated hybrid scaffolds with robust anti-osteoclastogenesis ability. Due to the synergistic effects of the LYN and HA components, the obtained three-dimensional hybrid scaffolds exhibited the dual effects of osteoclastic inhibition and osteogenic stimulation, thereby promoting bone tissue repair. Systematic characterization experiments confirmed the successful fabrication of LYN/HA-coated hybrid scaffolds, which exhibited an interconnected porous structure with nanoroughened surface topography, favorable hydrophilicity, and improved mechanical properties, as well as the sustained sequential release of LYN and Ca ions. In vitro experiments demonstrated that LYN/HA-coated hybrid scaffolds possessed satisfactory cytocompatibility, effectively promoting cell adhesion, spreading, proliferation, alkaline phosphatase activity, matrix mineralization, and osteogenesis-related gene and protein secretion, as well as stimulating angiogenic differentiation of endothelial cells. In addition to osteogenesis, the engineered scaffolds also significantly reduced osteoclastogenesis, such as tartrate-resistant acid phosphatase activity, F-actin ring staining, and osteoclastogenesis-related gene and protein secretion. More importantly, in a rat calvarial defect model, the newly developed hybrid scaffolds significantly promoted bone repair and regeneration. Microcomputed tomography, histological, and immunohistochemical analyses all revealed that the LYN/HA-coated hybrid scaffolds possessed not only reliable biosafety but also excellent osteogenesis-inducing and osteoclastogenesis-inhibiting effects, resulting in faster and higher-quality bone tissue regeneration. Taken together, this study offers a powerful and promising strategy to construct multifunctional nanocomposite scaffolds by promoting osteo/angiogenesis and suppressing osteoclastogenesis to accelerate bone regeneration.

## Introduction

Bone is a highly dynamic mineralized tissue with unique self-healing potential following mild injury. However, large-scale bone defects caused by traumatic injury, tumor excision, or infectious diseases may not heal spontaneously, inevitably leading to a significant economic and clinical impact^1^. It is estimated that approximately 5 billion dollars are required for bone defect repair annually in the United States alone, and a 10% annual increase in bone grafting procedures is expected^2^. Conventional bone defect management in the clinic includes bone transplants, with autografts being the “gold standard”; nevertheless, their usage is generally impeded by several drawbacks, such as limited graft sources, donor site morbidity, and additional surgical complications. On the other hand, allografts and xenografts are frequently associated with multiple potential risks, including immunogenic responses and host-donor junction complications, as well as risks of infection and disease transmission^3^. To overcome the abovementioned limitations and accelerate bone healing, researchers have tried various approaches to develop ideal bone graft substitute materials with enhanced physicochemical and biological properties to better emulate natural tissues, which would have significant clinical value^4,5^. Emerging biomaterial-based bone graft substitutes, which mimic the natural extracellular matrix (ECM), offer a promising therapeutic strategy for accelerating bone regeneration due to their inherent biocompatibility and superior biological performance, as well as great advantages in the delivery of bioactive agents and manipulation of stem cell fate^6,7^. In particular, biologically active molecules, including growth factors (large proteins, cytokines, hormones), genes, and small molecule drugs, exert considerable osteogenic bioactivity when combined with scaffolds^8^. Nevertheless, bone healing is a sophisticated and comprehensive process that involves not only osteogenesis but also osteoclastogenesis and angiogenesis. Natural bone possesses a variety of cells, including osteoblasts, osteoclasts, mesenchymal stem cells (MSCs), vascular endothelial cells, and different types of immune cells. Over the last few decades, a great deal of work has been done to promote bone healing through MSC-mediated osteogenesis in terms of enhanced proliferation and recruitment of MSCs, but the efficacy is suboptimal with high cost in clinical practice^9,10^. Osteoclast-related bone resorption may be a pivotal factor contributing to unsatisfactory bone repair efficacy. Recently, accumulating evidence has proven that osteoclasts play an indispensable role in bone formation, maintenance, and remodeling^11^. In addition, studies have pointed out that osteoclasts can resorb the bone matrix during bone metabolism and further release active transforming growth factor-β1 (TGF-β1) to recruit MSCs and induce osteogenesis^12^. During the bone mineralization process, osteogenesis without osteoclastic activity may suppress bone remodeling, which would impair the natural mechanism of bone repair and increase the risk of undesired bone overgrowth and osteosclerosis^9^. Therefore, to develop bone graft substitute materials with better performance, the most important and challenging task is to orchestrate osteogenesis and osteoclastogenesis, the two coupled processes during bone regeneration.

LYN-1604 (LYN), a recently developed small-molecule activator of UNC-51-like kinase 1 (ULK1), was first reported by Liu and Ouyang et al.^13^ in 2017. ULK1 is a mammalian serine/threonine kinase that normally binds to FIP200, ATG13, and ATG101 to form an autophagy initiation complex and mediate the autophagic reaction. Moreover, ULK1 has been proven to affect osteoclast formation and function by interfering with autophagy. Our recent study demonstrated that ULK1 was downregulated during osteoclast differentiation, and ULK1 downregulation correlated with osteoporosis, the most common osteometabolic disease characterized by low bone mass and deterioration of bone microarchitecture^14^. To further support the in vitro results, we established an osteoporosis model in ovariectomized mice and revealed that ovariectomized mice treated with LYN showed a higher bone mass, bone density, and numbers of trabeculae than ovariectomized mice without treatment. In light of this, ULK1 is a promising, specific, and potent therapeutic target for inhibiting osteoclastogenesis, thus enabling its targeted agonist LYN to be utilized as a small-molecule drug candidate for bone regeneration applications. However, due to the lack of a strong interaction between LYN and substrates, the application of LYN without proper delivery systems will suffer from several limitations, including poor physiological stability, rapid clearance (or burst release), nonspecific targeting, and low cell membrane permeability. To date, some physical and chemical methods, such as physical adsorption, chemical modification, grafting, plasma treatment, and other traditional surface modification techniques, have been developed to anchor molecules onto the substrate surface to exert their biological performance^15^. Because of the adhesive properties of polyphenols, mussel-inspired molecules, such as polydopamine (PDA), have been identified as effective agents for surface modification in biomedical applications^16^. The versatile adhesion properties, mild synthesis requirements, excellent biocompatibility, and facile immobilization of biomolecules make PDA coating widely applicable to improve interfacial integration between grafts and substrate surfaces, forming multifunctional organic/inorganic composite layers consisting of drugs, proteins, metal ions, apatites, and nanoparticles^17^. For example, Hasani-Sadrabadi et al. designed a series of nanofibrous membranes via the incorporation of a biomimetic polydopamine nanoscale coating, which can mimic the complex extracellular environment of periodontal tissue and serve as functional tissue constructs for periodontal regeneration. In addition, bone morphogenetic protein-2 (BMP-2)-derived peptides were further immobilized onto nanofibrous membranes via the self-polymerization of dopamine, which effectively improved the osteogenic potential of periodontal ligament stem cells in vitro^18^. Similar research has also been reported previously by Lee et al.^19^ and Ye et al.^20^. The delivery of osteogenic active agents such as BMP-2 is becoming an effective approach to accelerate bone healing; however, this design strategy focuses mainly on osteoinduction and the differentiation of osteoblasts without considering the influence of osteoclastogenesis on bone regeneration. Therefore, it is reasonable to conceive that the construction of bone repair materials that can simultaneously enhance osteogenesis and suppress osteoclastogenesis would be an effective strategy to promote bone tissue regeneration.

Despite the fascinating advantages concerning anti-osteoclastogenesis, LYN alone may intrinsically lack osteoinductive ability. Inspired by the mineralization processes in living organisms, numerous studies have proven that PDA coating has a strong capability to accelerate the mineral deposition of hydroxyapatite (HA), a bioceramic similar to the components of bone minerals, in the presence of simulated body fluids (SBFs)^21^. The abundant functional groups (catechol, amine, and imine) present in PDA can provide additional nucleation sites for HA deposition, thus accelerating the biomineralization process^18^. As the major inorganic portion of natural bone, HA is commonly used in orthopedic and dental materials and is capable of accelerating the bone defect healing process owing to its excellent biocompatibility, good osteoinductive nature, and chemical similarity to human bone. The deposition of bone-like HA nanocrystals covering graft substitutes can not only provide a favorable ECM-mimetic microenvironment for the adhesion, proliferation, and differentiation of osteogenesis-related cells (e.g., MSCs and osteoblasts) but also substantially enhance the osseointegration between implants and host bone tissues, thus improving the performance of scaffolds in bone tissue engineering applications^6,22^. Moreover, other studies have shown that HA has a high solubility in the physiological environment and can release nontoxic Ca ions to promote bone and blood vessel regeneration by activating calcium ion-sensing receptor signaling^23^. Therefore, with the help of the PDA-assisted technique as well as the complementary combination of LYN and HA, obtaining materials with the ability to facilitate osteogenesis and biomineralization while simultaneously inhibiting osteoclastogenesis will help to accelerate the repair and regeneration of bone defects.

**Fig. 1:**
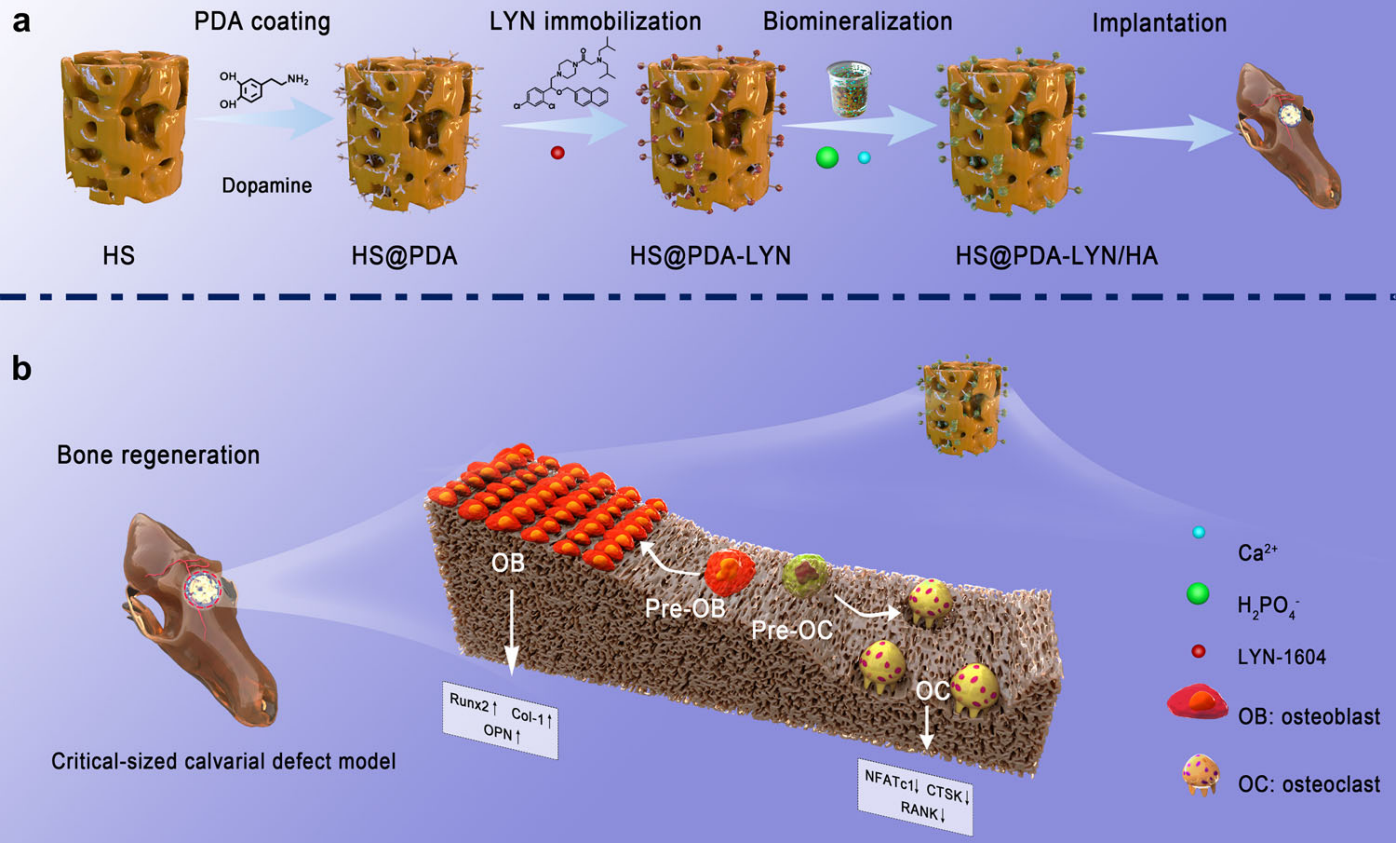
Schematic illustrations of the construction and application of multifunctional nanocomposite scaffolds. **a** The HS@PDA-LYN/HA scaffold was fabricated via a bioinspired PDA intermediate, followed by sequential immobilization of LYN and HA. **b** The dual biological function of HS@PDA-LYN/HA facilitated endogenous bone regeneration through osteogenic stimulation and osteoclastic inhibition.

Based on the aforementioned considerations, a dual therapeutic bone regeneration strategy with pro-osteogenesis and anti-osteoclastogenesis properties for bone repair was developed by the mussel-inspired PDA technique via the sequential immobilization of LYN and HA nanocrystals on a 3D porous substrate. Previously, our group developed a series of highly porous epichlorohydrin-crosslinked hydroxyethyl cellulose (HEC)/soy protein isolate (SPI) bicomponent scaffolds (HSs) that showed good biocompatibility and biodegradability both in vitro and in vivo^24–26^. For this reason, we selected biocompatible HS as the base part of the composite scaffold, followed by introducing a bioinspired polymerized PDA coating on its surface without complex and harsh chemical reactions. Upon creation of the PDA layer on the scaffold surface, the osteoclastic molecule (LYN) is immobilized on the PDA layer by Schiff base formation, and the combined effect of physical adsorption, electrostatic interaction, and strong chemical interaction between the PDA and tertiary amine of the LYN surface. Subsequently, the HA nanocrystals were precipitated on the networks of the obtained material (HS@PDA-LYN) through in vitro biomineralization, resulting in an off-the-shelf hybrid scaffold (HS@PDA-LYN/HA). Notably, the 3D porous and interconnected structure of HS not only enables sufficient PDA coating but also enhances the exposure of more functional groups, contributing to nucleation via chelation and HA growth on the scaffold surface. We hypothesized that the 3D hybrid nanocomposite system containing LYN and HA could promote osteogenic differentiation while suppressing osteoclastic differentiation in vitro, following the stimulation of robust bone regeneration in vivo due to the sustained release of bioactive Ca ions and LYN. To systematically test our hypothesis, the physicochemical structure and properties, hydrophilicity, in vitro LYN and Ca ion release behaviors, mechanical strength, and in vitro cytocompatibility of the fabricated scaffolds were investigated. Additionally, in vitro cell adhesion and osteogenic and osteoclastic differentiation were fully evaluated using mouse calvaria-derived MC3T3-E1 preosteoblastic cells and macrophages derived from mouse bone marrow (BMMs), respectively. The potential mechanism underlying the effects of LYN in biomaterial delivery for osteoclastic inhibition was also investigated. To further evaluate their bone regeneration performance in vivo, the hybrid scaffold was implanted into the critical-sized cranial defects of rats and subsequently analyzed by high-resolution microscopic computed tomography (micro-CT) scanning and histological examination at 4 and 8 weeks after implantation (Fig. 1). Overall, our results provide insight into the potential mechanism underlying the effects of 3D hybrid scaffolds in biomaterial delivery for osteoclastic inhibition and osteogenic promotion and develop an attractive therapeutic implantable biomaterial platform for functional bone regeneration.

## Results and discussion

### Design and fabrication of multifunctional nanocomposites

In the present experiment, a novel surface-modified HS 3D scaffold with LYN and HA was constructed via bioinspired dopamine chemistry. Figure 1 illustrates the construction of the LYN/HA-decorated multifunctional nanocomposite scaffolds and their dual biological functions (pro-osteogenesis and anti-osteoclastogenesis) through the sustained release of LYN and Ca ions to accelerate bone regeneration. Herein, we selected HS as the base part of the composite scaffold because of its 3D porous structure and favorable biocompatibility^27^, which were conducive to sufficient PDA coating. However, pristine HS scaffolds possess limited bioactivities to accelerate tissue regeneration by regulating cell behaviors ranging from viability to functionality. Drawing inspiration from mussel adhesive proteins that contain 3,4-dihydroxy-L-phenylalanine (L-DOPA), the as-prepared HS substrates were coated in a biomimetic fashion with PDA, causing the surface to become more versatile and functional. The introduction of PDA coating also proved to be helpful for cell adhesion and proliferation^28^. Nevertheless, this coating still lacks strong therapeutic functions to control osteogenesis and osteoclastogenesis, which greatly impedes its potential application in bone scaffolds. Studies have shown that the presence of PDA-coated structures provides favorable adhesive properties for the immobilization of functional molecules^29^. Moreover, the abundant functional groups (catechol, amine, and imine) of PDA coatings can offer more active sites for biomimetic mineralization. In this context, the immobilization of LYN and in situ biomineralization of HA nanocrystals proceeded sequentially on the PDA-coated surface via a two-step in situ deposition method, ultimately forming a multifunctional micro/nanostructured surface. Additionally, 10 × simulated body fluid (10 × SBF) was utilized in the study because it provides not only faster mineral formation but also a suitable supersaturated environment around the HEC/SPI matrix, thereby supplying the necessary clusters for the nucleation process and formation of amorphous calcium phosphate during the in vitro biomineralization process^30^.

### Characterization of multifunctional nanocomposites

**Fig. 2:**
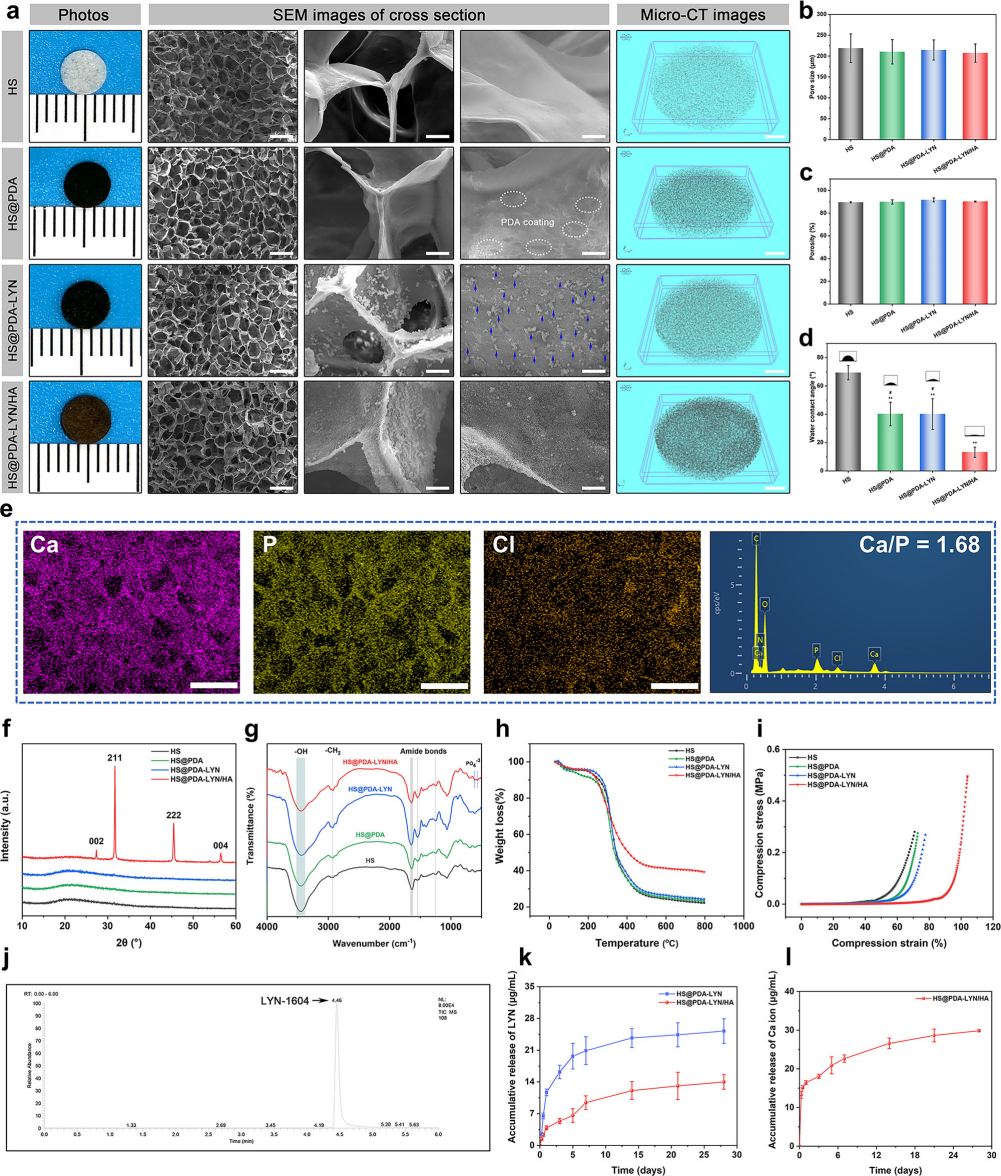
Morphology and characterization of surface-modified HS 3D scaffolds with LYN and HA via bioinspired dopamine chemistry. **a** Representative optical appearance and microstructure of different scaffolds after freeze-drying as observed by SEM and micro-CT. The blue arrows indicate the immobilization of LYN on the scaffold surface. **b** Average pore size, **c** porosity, and **d** WCA of the four kinds of scaffolds. **e** EDS mapping images and spectrum of HS@PDA-LYN/HA. **f** XRD spectra. **g** FTIR spectra. **h** TGA analysis of different scaffolds under a N2 atmosphere. **i** Representative stress–strain curve of scaffolds under compression. **j** Representative HPLC–MS/MS image of the HS@PDA-LYN/HA scaffold. **k** Accumulative LYN concentration released from HS@PDA-LYN/HA and HS@PDA-LYN/HA samples in Tris-HCl buffer solution. **l** Accumulative Ca ion concentration released from HS@PDA-LYN/HA samples in Tris-HCl buffer solution. Scale bar in **a**: from left to right 300, 20, 10 μm, 1 mm, in **e**: 250 μm. Data are expressed as the mean ± SD (*n* = 3). *P < 0.05 and **P < 0.01 indicate significant differences compared with the HS group. ^#^*P* < 0.05 and ^# #^*P* < 0.01 indicate significant differences compared with the HS@PDA-LYN/HA group.

The gross observation, surface morphology, and 3D microstructure of different scaffolds are displayed in Fig. 2a, in which the HS scaffold sample shows a light-yellow color. After PDA treatment, the general features of the HS@PDA and HS@PDA-LYN surfaces were notably changed with a homogeneous black coating. More meaningfully, the color of the resultant HS@PDA-LYN/HA progressively turned brown after in situ biomimetic mineralization, as shown in the optical images. To further investigate the effect of LYN and HA immobilization on scaffold architecture and physicochemical properties, we performed a series of comprehensive validation assessments in subsequent experiments. Surface and cross-sectional scanning electron microscopy (SEM) images of different scaffolds after freeze-drying are presented in Fig. 2a. Under low magnification, all scaffolds were characterized by a typical sponge-like structure consisting of highly interconnected macropores throughout the scaffolds, as demonstrated in our previous study^31^. These 3D porous and interconnected structures of scaffolds are important structural traits that significantly promote the ingrowth of new bone tissue, vascularization, and transportation of nutrients and waste material, thereby accelerating bone defect repair and regeneration^32^. Under high magnification, compared with HS substrates that showed relatively flat and smooth surfaces, the surface of HS@PDA substrates was rougher due to the deposition of PDA nanoparticles. On the other hand, from the SEM images of HS@PDA-LYN, it is clear that many small particles are uniformly distributed on the scaffold surface, which confirms the successful immobilization of LYN. After performing in situ biomineralization, the final resultant material featured many spherical mineral nanocrystals uniformly covering the scaffold skeleton without obvious aggregation. These results agree well with previous literature demonstrating that PDA coating can promote HA crystallization or biomineralization^33,34^. Additionally, the precipitation of inorganic HA nanocrystals throughout the network skeletons of the polymer matrix formed a continuous and stable mineralized layer (Supplementary Fig. 1), ultimately resulting in a nanoroughened surface topography. It was reported that HA-roughened surfaces tended to more effectively promote cell adhesion, spreading, proliferation, migration, and subsequent osteogenic differentiation than smooth surfaces^35^. Further analysis of the internal microarchitecture was obtained by micro-CT evaluation, which can display the 3D interconnected porous structure more clearly than SEM analysis. Interestingly, compared with the HS substrate, the architecture formed in HS@PDA, HS@PDA-LYN, and HS@PDA-LYN/HA, especially in HS@PDA-LYN/HA, had a higher local density, suggesting the uniform deposition of PDA and inorganic HA within the scaffold.

In addition to the 3D micro/nanostructured surface, the basic physical properties of different scaffolds were also characterized. It is well known that appropriate pore size and porosity are some of the critical parameters of bone repair scaffolds, which enable efficient nutrient/oxygen transportation and cell infiltration, as well as new bone tissue and blood vessel ingrowth^36^. SEM morphometric analysis indicated that the average pore sizes in the HS, HS@PDA, HS@PDA-LYN, and HS@PDA-LYN/HA groups were 218.8 ± 34.5, 210.4 ± 29.2, 214.6 ± 24.3, and 207.3 ± 21.9 μm, respectively, showing no significant difference among all the groups (Fig. 2b). On the other hand, the total porosities calculated by micro-CT in the HS, HS@PDA, HS@PDA-LYN, and HS@PDA-LYN/HA groups were 89.5 ± 0.5, 89.8 ± 1.9, 91.6 ± 1.8, and 90.2 ± 0.5%, respectively (Fig. 2c). It has been suggested that highly porous structures of 3D scaffolds facilitate cell adhesion and migration^3^. Both results indicated that the introduction of PDA coating, LYN, and HA did not significantly affect scaffold pore size and porosities, with all prepared scaffolds achieving a mean pore size of approximately 200 μm and porosity of approximately 90%. Evidence has shown that the optimal pore size for inducing angiogenesis and osteogenesis is between 100 and 500 μm, with a porosity of more than 80%^36^, which is in accordance with our current results. Furthermore, static water contact angle (WCA) measurements were performed to determine the surface hydrophilicity of different scaffolds. The smaller the WCA is, the better the hydrophilicity. As expected, a significant decrease in static WCA was observed on the surface of HS@PDA, HS@PDA-LYN, and HS@PDA-LYN/HA. As shown in Fig. 2d, on the surface of the HS substrates, the WCAs was (69.3 ± 5.1)°, while it was reduced to (40.1 ± 8.3)° and (40.1 ± 10.9)°, respectively, after PDA coating and LYN immobilization. Notably, the surface hydrophilicity of HS@PDA-LYN/HA (13.1 ± 3.7)° further improved after in situ mineralization. This phenomenon was mainly attributed to the introduction of PDA coating and HA, which is in line with previous work reporting the positive role of PDA and HA dually functionalized biomaterials on surface wettability^37–39^. The enhancement of surface hydrophilicity was proven to enable a profound effect on initial inflammation and subsequent biological performance between the implant surface and host surrounding tissue, including cell adhesion, proliferation, differentiation, and the formation of new bone and blood vessels^28,38^. Collectively, the combination of a 3D macroporous structure, PDA-mediated immobilization of LYN and HA, nanoroughened surface topography, and improved hydrophilicity made HS@PDA-LYN/HA more likely to exhibit a beneficial effect on promoting bone regeneration both in vitro and in vivo.

Subsequently, energy-dispersive spectroscopy (EDS) elemental mapping was used to determine the composition of different scaffolds, as illustrated in Fig. 2e and Supplementary Fig. 2a, b. For both HS@PDA-LYN and HS@PDA-LYN/HA samples, the major elements of C, N, and O exhibited homogeneous distribution on the scaffold surface, primarily originating from HS substrates. In addition, abundant Cl derived from LYN existed in the HS@PDA-LYN sample, indicating the successful immobilization of LYN. After biomineralization, elemental mapping images further showed that the Ca and P elements were homogeneously distributed alongside the porous skeleton, demonstrating the successful deposition of calcium phosphate (CaP) crystals, which was consistent with the SEM results. Analysis of the EDS spectrum further showed that HS@PDA-LYN/HA had a Ca/P atomic ratio (1.68) comparable to the stoichiometric ratio in HA, which was close to the ratio of 1.67 for natural bone tissue^33^. The formation of bone-like HA on the scaffolds should be attributed to the 3D porous structure and PDA coating, which provide a high specific surface area and bioactive surface containing functional groups, thus accelerating the efficacy of biomimetic mineralization^18^. More specifically, the surface catechol, amine, and imine groups of the bioactive PDA coating in the scaffold can attract Ca^2+^ cations of 10 × SBF and create a surface layer with a positive charge, which can then adsorb PO_4_^3−^ and make the HA nucleate and grow. Furthermore, the uniform deposition of CaP nanocrystals endowed HS@PDA-LYN/HA with enhanced biomineralization, favorable biocompatibility, and increased cell adhesion and proliferation. Previous studies have shown that a natural bone-like CaP coating (Ca/P ratio near 1.67) on biomaterial scaffolds could provide a biomimetic microenvironment for the proliferation, adhesion, and mineralization of osteoblasts by mimicking the mineralized interface of the native bone ECM^30,32^. Overall, EDS mapping of Ca, P, and Cl element distributions confirmed uniform and homogeneous CaP layers and LYN deposition in HS@PDA-LYN/HA.

To provide further evidence of surface modification, X-ray photoelectron spectroscopy (XPS) analysis was performed, as illustrated in Supplementary Fig. 2c–e. Although the HS and HS@PDA scaffold samples displayed similar levels of C, N, and O peaks, the results of high-resolution XPS spectra clearly showed a shift of the N 1 s peak from 400.1 to 400.2 eV in the HS@PDA scaffold group owing to the presence of primary amines in the PDA layer. Likewise, a noticeable change in carbon bonds (C 1 s) was also detected in the high-resolution carbon spectra. The intensity of the C−N/C−O peak significantly increased in the HS@PDA scaffold group, proving the presence of PDA. After the immobilization of LYN, it can be found that HS@PDA-LYN samples have a visible Cl 2p peak (199.2 eV), which is in line with the EDS results, indicating that LYN was successfully loaded on the surface of HS@PDA samples. For HS@PDA-LYN/HA, Ca 2p (347.6 eV), P 2p (133.4 eV), and Cl 2p (199.2 eV) peaks appeared, indicating efficient PDA-mediated immobilization of LYN and HA on the surface of HS@PDA-LYN/HA.

To identify the phase compositions of the deposited mineral layer, X-ray diffraction (XRD) analysis was performed on various scaffolds, and the results are illustrated in Fig. 2f. Specifically, there was nearly no difference detected from the XRD spectra of HS, HS@PDA, and HS@PDA-LYN, implying that the surface modification of HS with PDA and LYN did not influence the crystal structure of HS substrates. After SBF immersion, as expected, several characteristic diffraction peaks at 27.3°, 31.7°, 45.4°, and 56.5° were newly detected in HS@PDA-LYN/HA, which were assigned to the (002), (211), (222), and (004) crystalline planes, respectively. These diffraction peaks showed good agreement between the peaks of the formed minerals and those of standard HA (JCPDS No. 74-0566), confirming that the precipitated mineral is HA^35^. We then performed Fourier transform infrared (FTIR) spectroscopy to characterize the functional groups of different scaffolds, as displayed in Fig. 2g. All scaffold groups showed a characteristic amide I peak (1646 cm^−1^), amide II peak (1548 cm^−1^), and amide III peak (1247 cm^−1^), which are representative of the secondary structure of amino acids in SPI. The presence of broad peaks at 3446 and 2925 cm^−1^ corresponding to the stretching vibrations of –OH and –CH, respectively, could be attributed to the HEC component, which agrees well with results from previous studies on HS-based biomaterials^30^. After in situ mineralization, the FTIR spectrum of the final nanocomposite HS@PDA-LYN/HA showed some differences from those of other scaffold samples. The characteristic peaks of crystalline phosphate (PO_4_^3−^) at 607 and 566 cm^−1^ were detected in the FTIR spectra, confirming the formation of HA nanocrystals, which are in line with the SEM, EDS, XPS, and XRD results.

The thermal stability and content of precipitated minerals of different scaffolds were quantified by TGA. As shown in Fig. 2h, a slight weight loss (approximately 10 wt%) was observed between 30 and 180 °C in all samples. This may be responsible for the evaporation of physically absorbed water. Subsequently, a drastic drop in scaffold content was observed, which was assigned to the decomposition of the organic components of scaffolds. Interestingly, TGA curves demonstrated that HS, HS@PDA, and HS@PDA-LYN had similar weight losses, which began at approximately 290 °C, achieving complete degradation at 650 °C, resulting in a remaining weight ≈23 wt%. For the HS@PDA-LYN/HA group, the residual weight at 800 °C was 39.3 wt%, which was 16.3% higher than that of other scaffolds, representing the actual HA content of HS@PDA-LYN/HA. Altogether, the TGA degradation profiles further confirmed the successful introduction of HA, which significantly enhanced the thermal stability of the HS@PDA-LYN/HA nanocomposites.

To develop a desirable biomaterial scaffold for bone regeneration, the material should provide sufficient structural support and share the load with the nearby bone tissues during the bone repair period^36^. Therefore, we conducted a mechanical compression test on different scaffolds. Figure 2i shows the representative compression stress–strain curves and the mechanical properties of various scaffolds. The results indicated that all scaffolds exhibited a certain level of stiffness and deformation resistance under aqueous conditions. Compared to the pure HS sample, the maximum compressive strengths of HS@PDA, HS@PDA-LYN, and HS@PDA-LYN/HA exhibited dramatic improvements with the introduction of the PDA coating and HA. Notably, HS@PDA-LYN/HA possessed the maximal compressive strength among all groups, which may be due to the synergistic reinforcement of the PDA coating and mineralized HA nanocrystals, thus improving the matrix stiffness. These findings were in good agreement with a previous report that the organic–inorganic integrity and uniform layer of HA are advantageous to improving the mechanical properties of tissue engineering scaffolds^40^. Additionally, the improved mechanical strength of the mineralized scaffolds by introducing HA nanocrystals was beneficial in supporting the microenvironment of bone defects undergoing the repair process and facilitating the growth of new bone tissue^41^. The above results supported the conclusion that the immobilization of LYN and HA on the HS substrate network was successful, and the functionalized HS@PDA-LYN/HA had the expected 3D microstructure and physicochemical properties.

Figure 2j–l shows the cumulative release curve of LYN and Ca ions in Tris-HCl buffer over 28 days. In this study, tris-buffer was used due to its neutral pH and high buffering capacity without any intervention of ion components. Both HS@PDA-LYN and HS@PDA-LYN/HA could release LYN over 28 days in a sustained manner, as measured by high performance liquid chromatography–tandem mass spectrometry (HPLC–MS/MS). In total, the LYN concentration increased gradually from both HS@PDA-LYN and HS@PDA-LYN/HA with time; however, the LYN concentration in HS@PDA-LYN/HA was lower than that in HS@PDA-LYN within the testing time, indicating that the prepared HS@PDA-LYN/HA had the ability to sustain the long-term release of LYN and to enhance the stability of pure HS@PDA-LYN. This effect was possibly attributed to the barrier effect of the uniform and homogeneous HA coating on the scaffold surface. Furthermore, sustained release of Ca ions from the HS@PDA-LYN/HA group was detected, with the cumulative release increasing from 16.4 ± 0.5 µg/mL after 1 day of immersion to 29.8 ± 0.2 µg/mL after 28 days of immersion. According to previous studies, Ca ions play a vital role in osteogenesis, while suitable concentrations of Ca also stimulate angiogenesis in vitro and in vivo^42,43^. Thus, stable and continuous release of LYN and Ca would provide powerful conditions for osteoclastic inhibition and osteogenic stimulation both in vitro and in vivo.

### In vitro cytocompatibility

**Fig. 3:**
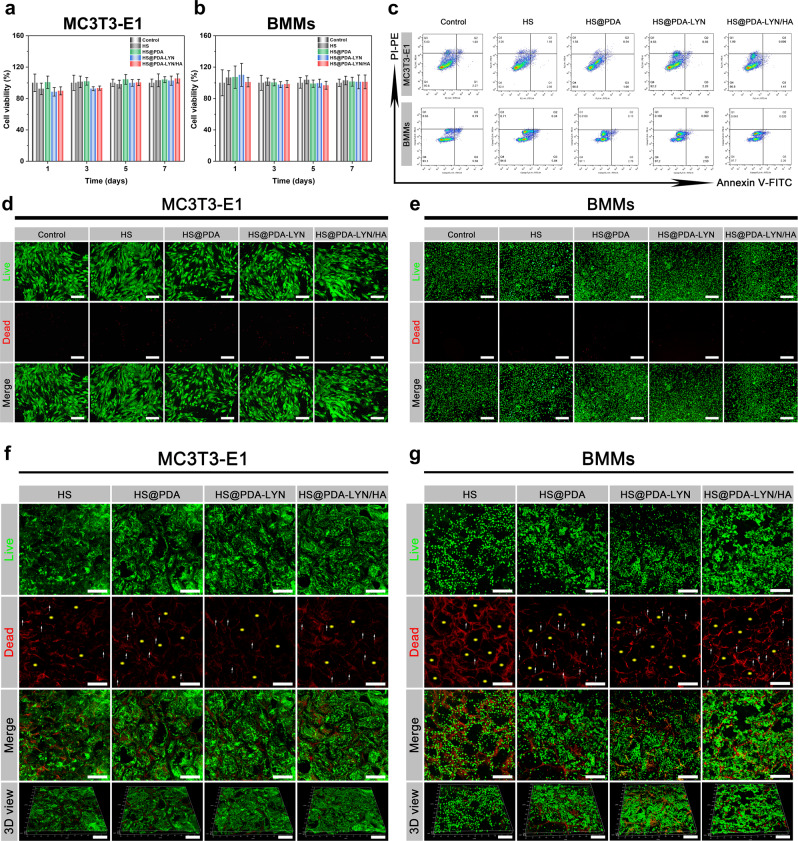
Cytocompatibility of the surface-modified HS 3D scaffold with LYN and HA in vitro. Cell viability of **a** MC3T3-E1 cells and **b** BMMs detected using the CCK-8 assay after culturing with scaffold extracts for 1, 3, 5, and 7 days. **c** Representative dot plots of annexin-V and PI double-stained MC3T3-E1 cells and BMMs following various treatments. Annexin V-FITC was used to identify early apoptotic cells by showing green fluorescence, and PI was used to identify late apoptotic cells by showing red fluorescence. Q1 represents the percentage of necrotic cells, Q2 represents the percentage of late apoptotic cells, Q3 represents the percentage of early apoptotic cells, and Q4 represents the percentage of live cells. Representative fluorescence images of live/dead staining of **d** MC3T3-E1 cells and **e** BMMs cocultured with different scaffold extracts for 3 days. Representative confocal fluorescence images of live/dead staining of **f** MC3T3-E1 cells and **g** BMMs cultured on scaffold surfaces for 3 days. The live cells were stained green, and dead cells were stained red. The yellow asterisks indicate the 3D porous structure of the fabricated scaffolds. The white arrows indicate dead cells. Scale bar in **d**, **e**: 200 μm, in **f**, **g**: 250 μm. Data are expressed as the mean ± SD (*n* = 3).

To achieve successful bone regeneration, bone-implant materials should be nontoxic or slightly toxic and show favorable cytocompatibility. Here, we evaluated the cytocompatibility of different samples using MC3T3-E1 preosteoblastic cells and BMMs as model cells due to their well-established protocols for in vitro cell culture experiments^37,44^. The cell surface markers first assessed by flow cytometry indicated that the obtained BMMs expressed high percentages of CD11b (approximately 96.6%, Supplementary Fig. 3), confirming the characteristic surface markers of macrophages. Then, the metabolic activity of various scaffold extracts to MC3T3-E1 cells and BMMs at 1, 3, and 7 d was investigated using the Cell Counting Kit-8 (CCK-8) assay. As shown in Fig. 3a, b, there was no significant difference in cell viability between the control, HS, HS@PDA, HS@PDA-LYN, and HS@PDA-LYN/HA groups at all time points. To support the CCK-8 results, flow cytometry was performed after 3 days of culture in the extracts of different scaffolds. The results revealed that both MC3T3-E1 cells and BMMs maintained a high survival rate above 94% and a negligible apoptosis rate (Fig. 3c and Supplementary Fig. 4). The same trend was observed with live/dead staining, verifying the high cell viability of both MC3T3-E1 cells and BMMs after receiving similar treatment (Fig. 3d, e). These findings were consistent with the results of previous studies, which indicated the excellent cytocompatibility of HS-based biomaterials^25,26^ and further confirmed the notion that the engineered surfaces modified with PDA, LYN, and HA did not induce significant cytotoxicity.

In addition to guaranteeing the cytocompatibility of the scaffold extracts, we also tried to seed the cells directly onto the scaffold surfaces and then imaged them with confocal Z-stacks. As shown in Fig. 3f, g, the live/dead-stained images of both MC3T3-E1 cells and BMMs on HS, HS@PDA, HS@PDA-LYN, and HS@PDA-LYN/HA scaffolds revealed that nearly all the cells were alive and uniformly distributed within the 3D porous structure of the scaffold, showing a desirable growth status. The cytocompatibility of direct contact reflected by the CCK-8 assay revealed a similar result, which is shown in Supplementary Fig. 5. The OD values of the HS@PDA, HS@PDA-LYN, and HS@PDA-LYN/HA groups increased gradually with incubation time and exhibited a similar tendency to the HS control group during the coculture period; however, there was no significant difference among the four groups. This positive effect on cytocompatibility may be related to the combined advantages of the PDA coating, 3D porous microstructures, high porosity, nanoroughened surface topography, suitable mechanical strength, and excellent hydrophilicity, providing a favorable microenvironment for cell attachment, survival, and proliferation^39^. In addition, some studies have also shown that direct cell-substrate interactions play a significant modulatory role in cell fate and function because they can directly influence cell proliferation and differentiation^45^. In summary, the results from both direct and indirect evaluations verified that all scaffolds and their leaching degradation products were relatively “green” and possessed good cytocompatibility.

**Fig. 4:**
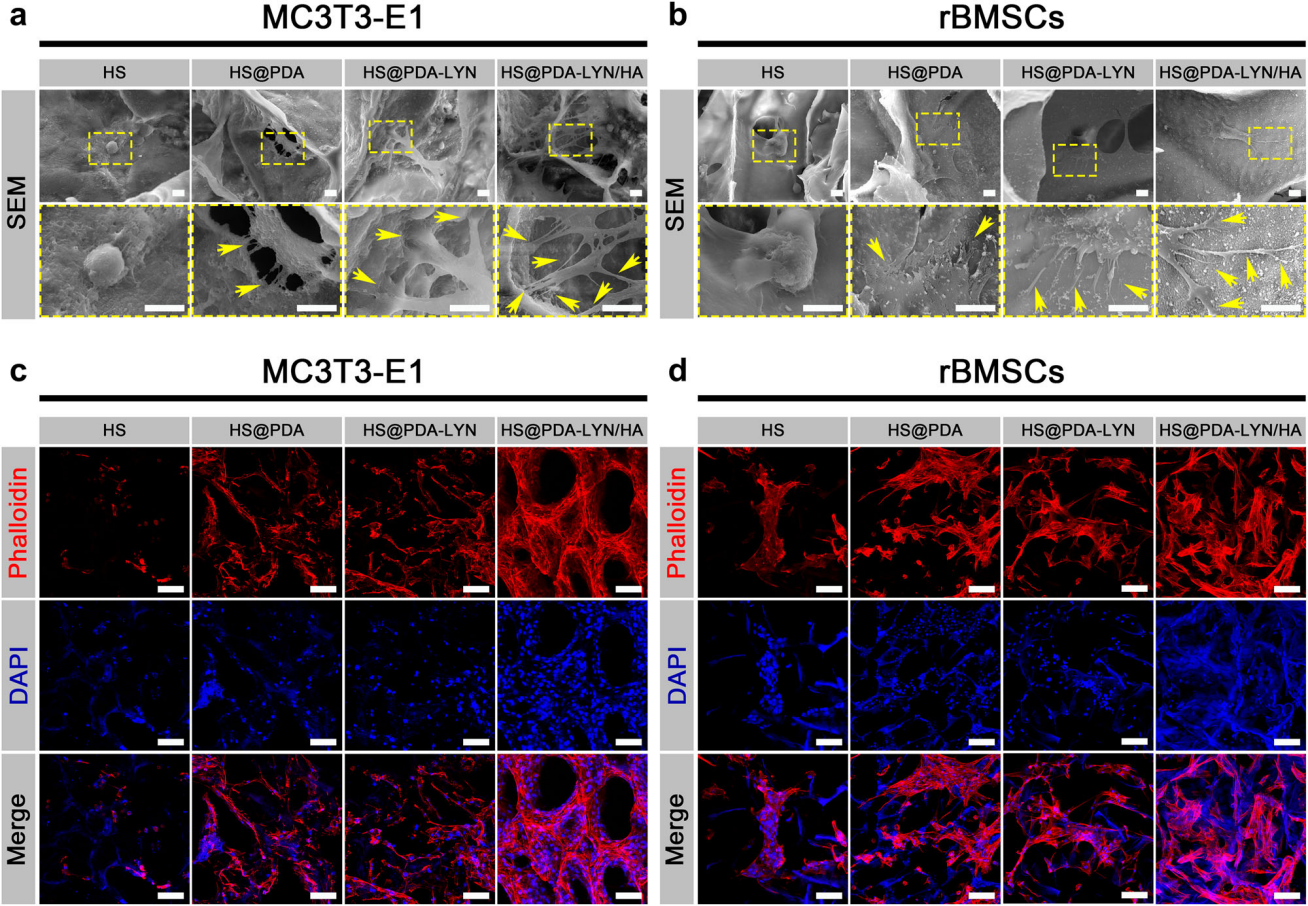
Cell adhesion, spreading and morphology on different samples. Representative SEM images of **a** MC3T3-E1 cells and **b** rBMSCs cultured on different scaffolds after 7 days. Representative confocal fluorescence images of F-actin (red) and nuclei (blue) in **c** MC3T3-E1 cells and **d** rBMSCs cultured on different scaffolds after 7 days. The yellow arrows indicate filopodia of cells adhered to scaffold surfaces. Scale bar in **a**, **b**: 10 μm, in **c**, **d**: 100 μm.

To further investigate the cell adhesion and spreading morphology on different scaffold samples, SEM and confocal laser scanning microscopy (CLSM) observations were performed. As shown in Fig. 4a, there was considerable variation in cellular adhesion and spreading morphology for MC3T3-E1 cells cultured on various scaffolds. Specifically, the cells on the HS substrate presented a near-spherical cellular shape morphology with a few protrusions, implying poor cell adhesion. In contrast, the MC3T3-E1 cells cultured on HS@PDA, HS@PDA-LYN, and HS@PDA-LYN/HA had a well-spreading morphology and closely adhered to the scaffold surface with numerous pseudopodia, indicating good attachment and spreading. In particular, the cells on HS@PDA-LYN/HA exhibited a highly branched osteoblastic-like morphology with elongated filopodia and pseudopodia, resulting in a more intimate interaction with the substrate matrix (Supplementary Fig. 6). It is widely accepted that the surface physicochemical properties of substrates govern cell behavior and thus master tissue regeneration. Additionally, osteoblasts are highly receptive to the surface chemistry, roughness, and hydrophilicity of biomaterial scaffolds^46^. In this regard, the strong adhesion of elongated filopodia and pseudopodia on the HS@PDA-LYN/HA scaffold can possibly be ascribed to the strong interactions of the filopodia with the HA-coated nanostructured surface as well as increased surface hydrophilicity. This finding is consistent with previously published literature for HA-coated material in terms of cells producing filamentous extension with elongated cell morphology^47^.

The morphologies of cells cultured on different scaffolds were further evaluated by phalloidin/4′,6-diamidino-2-phenylindole (DAPI) staining. As shown in Fig. 4c and Supplementary Fig. 7, MC3T3-E1 cells spread well and generated more mature F-actin on HS@PDA, HS@PDA-LYN, and HS@PDA-LYN/HA compared with those cultured on the HS substrate. Interestingly, the actin filaments in MC3T3-E1 cells on the HS@PDA-LYN/HA scaffold possessed numerous branched stretching directions with long filopodia, probably due to the interconnected porous structure, bone-like HA nanocrystals, increased roughness and hydrophilicity on the surface, providing abundant anchors for cell attachment and spreading. The SEM and CLSM images of rat bone marrow-derived mesenchymal stem cells (rBMSCs) cultured on various scaffolds showed a similar phenomenon (Fig. 4b, d). The results showed that rBMSCs seeded on the HS@PDA-LYN/HA scaffold exhibited a healthier morphology and spread better than those seeded on other scaffold surfaces, exhibiting evident 3D growth and uniform distribution. Therefore, HS@PDA-LYN/HA could facilitate cell stretching and attachment with long stretching distances, which was essential for the proliferation and further differentiation of MSCs for bone regeneration applications^48^. Based on these results, it is reasonable to speculate that the highly interconnected porous structure together with the presence of nanostructured HA ultimately increased the surface bioactivity and therefore facilitated the cellular interaction^49^. In addition, another key factor affecting cell adhesion is the ECM protein adsorbed on the substrate surface, including fibronectin, vitronectin, and other signaling molecules^24^. In particular, the binding of proteins to substrate surfaces occurs prior to cell attachment and is dominated by surface physicochemical properties, e.g., surface microstructure, roughness, and wettability. Significant research efforts have proven that biomimetic HA coatings can facilitate the adsorption of some bioactive ions and adhesive proteins, leading to the diffusion of cytoskeletal actin filaments on the substrate and ultimately mediating cell differentiation and mineralization^50^. This may provide an explanation for the favorable adhesion and F-actin assembly of both MC3T3-E1 cells and rBMSCs in the present study. Furthermore, it has been documented that cell morphology plays a pivotal role in regulating the cell phenotype, and a spindle-shaped morphology is beneficial for promoting cell behaviors by targeting the proliferation and differentiation of osteoblasts^46^. These results collectively suggested that the prepared HS@PDA-LYN/HA enabled a superior 3D microenvironment for cell growth, proliferation, and differentiation through their characteristic surface properties, thus exhibiting great potential as a biocompatible platform for in vitro cell culture.

### In vitro osteogenesis

**Fig. 5:**
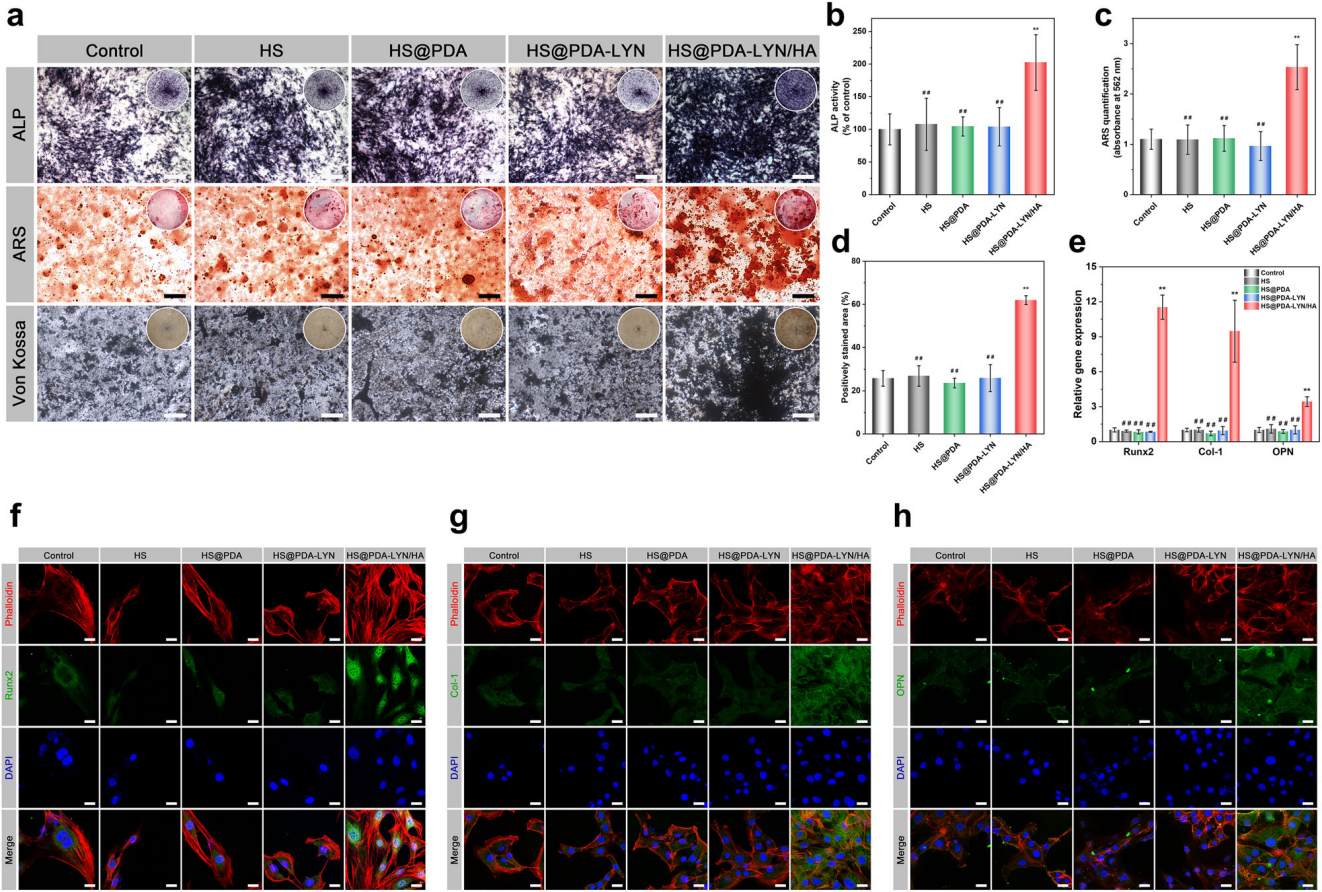
In vitro osteogenic differentiation behaviors of MC3T3-E1 cells after different treatments. **a** Representative ALP staining, ARS staining, and Von Kossa staining assays for MC3T3-E1 cells incubated with different scaffold extracts for 7, 14, and 21 days. Quantitative analysis of **b** ALP activity, **c** ARS staining, and **d** Von Kossa staining in different groups. The insets depict the digital images. Cells cultured without adding scaffold extracts served as a negative control. **e** The expression of osteogenesis-related genes, including Runx2, Col-1, and OPN, as determined by qRT–PCR assay. Representative immunofluorescent staining images of **f** Runx2 (green), **g** Col-1 (green), and **h** OPN (green) in MC3T3-E1 cells incubated with different extracts for 7 days. F-actin and cell nuclei were labeled with fluorescent red and blue, respectively. Images were captured using confocal microscopy. Scale bar in a: 200 μm, in **f**–**h**: 25 μm. Data are expressed as the mean ± SD (*n* = 3). *P < 0.05 and **P < 0.01 indicate significant differences compared with the control group. ^#^*P* < 0.05 and ^##^*P* < 0.01 indicate significant differences compared with the HS@PDA-LYN/HA group.

Encouraged by the above results, alkaline phosphate (ALP) activity, Alizarin red S (ARS) staining, and Von Kossa staining assays were performed to verify the osteogenic differentiation of MC3T3-E1 cells induced by different scaffolds. Typically, ALP is a key early-stage indicator of osteogenic differentiation, whereas extracellular matrix calcium deposition is considered a late-stage biochemical marker of osteogenesis^47^. As shown in Fig. 5a, compared with the control, HS, HS@PDA, and HS@PDA-LYN scaffolds, the HS@PDA-LYN/HA scaffold group had more ALP-positive cells, as evidenced by the presence of the strongest bluish intensity, suggesting the good osteogenic capacity of HS@PDA-LYN/HA. Quantitative analysis showed that the ALP activity levels were nearly 1.9-fold higher in the HS@PDA-LYN/HA scaffold group than in all other groups (Fig. 5b). A similar trend was observed in the ARS and Von Kossa staining assays (Fig. 5a), with MC3T3-E1 cells in the HS@PDA-LYN/HA group exhibiting abundant induced mineralized nodules, while only some small mineralized nodules were observed in all other groups. The quantitative analysis of the mineralized matrix also agreed with the corresponding staining, suggesting that the HS@PDA-LYN/HA scaffold group had the most substantial mineralization ability among all groups (Fig. 5c, d). The enhancement of both early and late osteogenic differentiation was mainly attributed to the moderate Ca ion concentrations released by HS@PDA-LYN/HA, which may function as critical biochemical signals to stimulate cell behavior^23^. This finding was consistent with previous studies, which suggested that the sustained delivery of therapeutic bioactive ions, such as Ca, Zn, Sr, and Si ions, was significantly associated with cellular functions^22,51^. Interestingly, rBMSCs treated with various extracts also showed a trend similar to that of MC3T3-E1 cells in terms of ALP, ARS, and Von Kossa staining (Supplementary Fig. 8), confirming the improved capability for in vitro osteogenesis of HS@PDA-LYN/HA through sustained Ca ion release. Except for the HS@PDA-LYN/HA groups, the ALP activity and calcium deposition did not exhibit significant differences among the control, HS, HS@PDA, and HS@PDA-LYN groups, implying that the immobilization of LYN did not adversely affect the osteogenic differentiation of MC3T3-E1 cells. Subsequently, the expression levels of osteogenesis-related genes, including Runt-related transcription factor 2 (Runx2), type I collagen (Col-1), and osteopontin (OPN), were examined using a quantitative real-time polymerase chain reaction (qRT–PCR) assay. Runx2 is an essential osteogenic transcription marker and is expressed in the early stage of osteogenic differentiation. It can activate the transcription and expression of ALP, OPN, Col-1, and relevant genes^37^. As shown in Fig. 5e, after culturing for 7 days, all osteogenesis-related genes were distinctively upregulated in MC3T3-E1 cells from the HS@PDA-LYN/HA group compared with those incubated in the extract from all other groups. More specifically, the expression levels of Runx2, Col-1, and OPN in MC3T3-E1 cells increased to 11.5-, 9.5-, and 3.4-, respectively, in the HS@PDA-LYN/HA group compared with the control group, while no significant difference was detected for all genes among the control, HS, HS@PDA, and HS@PDA-LYN groups. To further validate the obtained results, the expression levels of the corresponding proteins were investigated by immunofluorescent staining. As displayed in Fig. 5g, h, MC3T3-E1 cells treated with the HS@PDA-LYN/HA extract were characterized by the most abundant positive protein expression, implying enhanced osteogenic differentiation of MC3T3-E1 cells. These favorable outcomes should be attributed to the in situ-formed HA, resulting in the continuous release of Ca ions (Fig. 2i), which, in turn, stimulated cellular mineralization and differentiation in vitro by promoting the production of endogenous osteogenic factors (ALP, Runx2, etc.). Previous studies have elucidated the mechanism by which bioactive Ca ions regulate osteogenesis-related genes, including Runx2, Col-1, and OPN, inside osteoblasts^22^. Moreover, the effects of bioactive ions on regulating cellular functions in osteogenesis have also been well documented by other researchers^51,52^. A recent study also reported that HA-functionalized biomaterials could promote the expression of Runx2, inducing a higher level of osteogenic differentiation in vitro^37,40^. These results clearly demonstrated that HS@PDA-LYN/HA and its biodegradation products significantly promote the differentiation of preosteoblasts, verifying its desirable osteoinductive property.

### In vitro anti-osteoclastogenesis

**Fig. 6:**
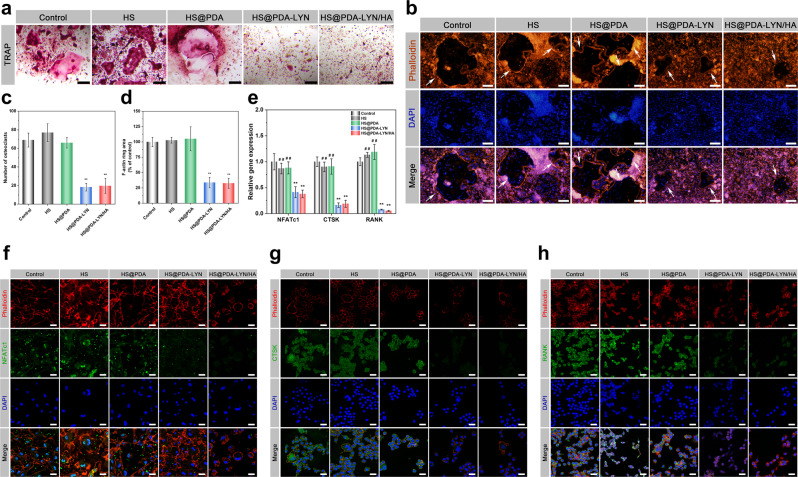
In vitro osteoclastic differentiation behaviors of BMMs after different treatments. Representative **a** TRAP staining and **b** F-actin ring staining assays for BMMs incubated with different scaffold extracts. The white arrows indicate the representative sealing zone of osteoclasts. Quantitative analysis of **c** TRAP staining and **d** F-actin rings in different groups. **e** The expression of osteoclastogenesis-related genes, including NFATc1, CTSK, and RANK, as determined by qRT–PCR assay. Representative immunofluorescent staining images of **f** NFATc1 (green), **g** CTSK (green), and **h** RANK (green) in BMMs incubated with different extracts. F-actin and cell nuclei were labeled with fluorescent red and blue, respectively. Images were captured using confocal microscopy. This suggested that the newly developed LYN-containing scaffolds may inhibit the osteoclastic differentiation of BMMs by sustaining the supply of LYN. Scale bar in a: 50 μm, in **b**: 200 μm, in **f**–**h**: 25 μm. Data are expressed as the mean ± SD (*n* = 3). *P < 0.05 and **P < 0.01 indicate significant differences compared with the control group. ^#^*P* < 0.05 and ^# #^*P* < 0.01 indicate significant differences compared with the HS@PDA-LYN/HA group.

As mentioned in the previous sections, we observed that both HS@PDA-LYN and HS@PDA-LYN/HA could effectively release LYN in a sustained manner, and the in situ mineralization of the HA coating could be further used to mitigate LYN release (Fig. 2k). In this section, we present the evaluation of the effects of LYN on osteoclast formation, F-actin ring immunofluorescence, and tartrate-resistant acid phosphatase (TRAP) staining, as well as gene and protein expression related to osteoclastic activity. As shown in Fig. 6a, b, TRAP staining revealed that osteoclastic enzymatic activity was substantially reduced in the HS@PDA-LYN and HS@PDA-LYN/HA groups, with a significantly decreased number of TRAP-positive multinucleated osteoclasts, indicating the inhibition of osteoclast formation (Fig. 6c). Next, the formation of an actin ring, a typical actin structure essential for bone resorption by active osteoclasts, was visualized by fluorescence microscopy. Interestingly, the results from phalloidin immunofluorescent staining showed that the F-actin rings were limited and localized in the HS@PDA-LYN and HS@PDA-LYN/HA groups (Fig. 6d), which might be attributed to the sustained release of LYN from the hybrid scaffolds. This was corroborated by the results of the comparison of the control and other experimental groups, suggesting that LYN may be the most important reason that the hybrid scaffolds inhibited osteoclastogenesis.

As is widely known, osteogenesis and osteoclastogenesis are two crucial processes for bone regeneration. According to our recent study, ULK1 may act as a critical mediator in accelerating bone regeneration by regulating osteoclastic activity^14^. Specifically, ULK1 was downregulated during the osteoclast differentiation process, and treatment with its activator LYN significantly inhibited osteoclastogenesis and indirectly promoted osteogenesis. As shown in Supplementary Fig. 9, both qRT–PCR and western blotting assays confirmed that the expression of ULK1 was significantly decreased during the osteoclast differentiation process, which was consistent with our previous results^14^. To further elucidate the effect of LYN-containing scaffolds on osteoclastogenesis, the expression of osteoclastogenesis-related genes, including nuclear factor of activated T cells 1 (NFATc1), cathepsin K (CTSK), and receptor activator of NF-κB (RANK), three main regulatory factors of osteoclastogenesis, was detected by qRT–PCR assay. As illustrated in Fig. 6e, the expression of NFATc1, CTSK, and RANK was significantly downregulated by the treatment of conditioned medium with LYN-containing scaffolds, indicating that the extract solution reduced the osteoclastic differentiation of BMMs. Immunofluorescence staining of NFATc1, CTSK, and RANK yielded results that were similar to those obtained by qRT–PCR assay. As displayed in Fig. 6f–h, the fluorescence intensity of both the HS@PDA-LYN and HS@PDA-LYN/HA groups decreased significantly, while no significant change was detected in the control and other experimental groups. The robust inhibitory effects on osteoclastic differentiation and functional activity should be attributed to the sustained release of LYN from HS@PDA-LYN and HS@PDA-LYN/HA. It has been reported that LYN can suppress osteoclastic differentiation by regulating cell autophagy through activating the AMPK signaling pathway, which plays a vital role in regulating cell metabolism and differentiation^53^. Altogether, these results supported our hypothesis that the immobilization of LYN and HA nanocrystals on porous scaffolds via bioinspired dopamine chemistry could effectively inhibit osteoclastogenesis and promote osteogenesis.

### In vitro angiogenesis

**Fig. 7:**
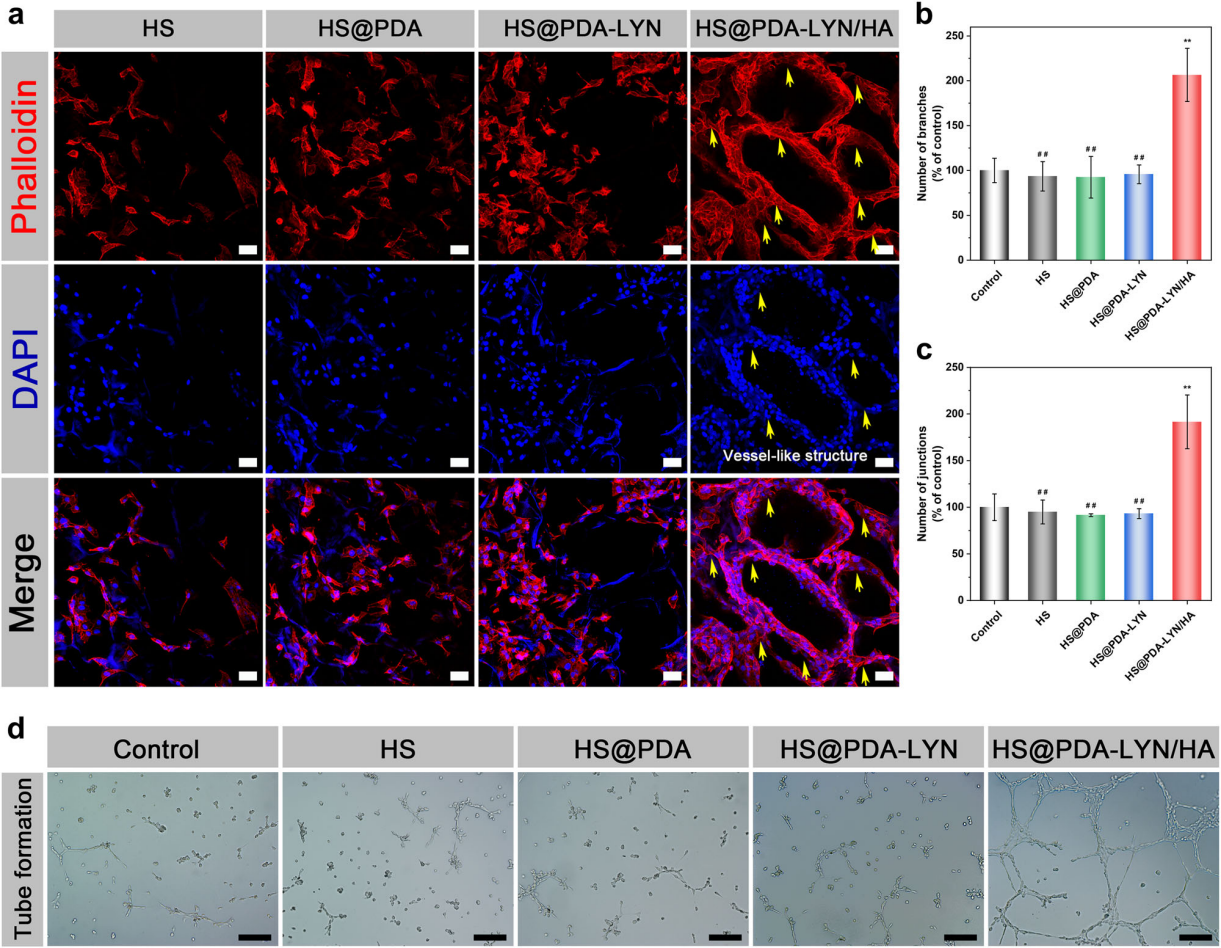
In vitro angiogenic differentiation behaviors of HUVECs after different treatments. **a** Representative confocal fluorescence images of F-actin (red) and nuclei (blue) in HUVECs cultured on different scaffolds for 14 days. The yellow arrows indicate the vessel-like structure. **b**–**d** Representative tube formation images for HUVECs incubated with different extract samples and quantitative analysis of the tube formation ability in different groups. Scale bar in **a**: 50 μm, in **d**: 200 μm. Data are expressed as the mean ± SD (*n* = 3). *P < 0.05 and **P < 0.01 indicate significant differences compared with the control group. ^#^*P* < 0.05 and ^# #^*P* < 0.01 indicate significant differences compared with the HS@PDA-LYN/HA group.

Along with osteogenesis and osteoclastogenesis, the angiogenic differentiation of endothelial cells is another crucial factor for accelerating bone regeneration, as sufficient blood supply favors nutrition exchange, progenitor cells, and circulating factor delivery^10^. Previous research has shown that the chemical composition of calcium phosphates influences neovascularization, and HA-functionalized biomaterials can substantially improve angiogenesis, leading to enhanced angiogenic activity^52,54^. In this study, the effect of different scaffolds on the actin cytoskeleton of human umbilical vein endothelial cells (HUVECs) was examined. As shown in Fig. 7a, HUVECs spread well on each sample, and more cells were attached to the HS@PDA-LYN/HA surface, which displayed mature filamentous F-actin protein due to the synergistic stimulation of surface nanotopography and Ca ion release. More interestingly, with the help of the formed macropores and nanoroughened surface topography, cells cultured on the HS@PDA-LYN/HA scaffold surface acted as climbers adhering along the scaffold skeleton, which were triggered to assemble into blood vessel-like structures on its surface within 14 days of incubation. The blood vessel-like arrangement of cells and actin on HS@PDA-LYN/HA was mainly ascribed to classical contact guidance theory^55^, suggesting that the HS@PDA-LYN/HA scaffold could provide an outstanding biomimetic microenvironment for the angiogenesis of HUVECs even without the addition of Matrigel.

In the subsequent tube formation assay, cells were further treated with extract liquid of scaffolds to study the vascularization process of HUVECs. As shown in Fig. 7d, HUVECs in the control, HS, HS@PDA, and HS@PDA-LYN extracts were separated from each other, and only a few fragile tubes were observed, showing no significant difference. In sharp contrast, the cells cultured in HS@PDA-LYN/HA revealed more capillary-like networks with favorable morphological characteristics. Correspondingly, the quantitative analysis results of the number of branches and junctions also supported that HS@PDA-LYN/HA was beneficial to improving the tube formation ability (Fig. 7b, c), and similar results have also been reported in previous literature^51^. Furthermore, after incubation for 7 days, the expression of angiogenic proteins was investigated by immunofluorescence staining of CD31, a specific marker of endothelial cells. As shown in Supplementary Fig. 10, the fluorescence intensity of CD31 in the HS@PDA-LYN/HA group was significantly stronger than that in the other groups, indicating that there was more CD31 protein expression. In a previous study, the biodegradation products of HA released various ions (mainly Ca ions) and had the potential to induce angiogenesis by promoting the production of endogenous angiogenic factors (VEGF, bFGF, etc.), which is conducive to the process of bone healing and repair^23^. In addition, it is worth mentioning that LYN immobilization did not adversely affect the in vitro angiogenesis of HUVECs. According to the above results of all experiments, we believe that the nanocomposite system containing LYN and HA exhibits superior capacities to induce osteogenesis and angiogenesis while inhibiting osteoclastogenesis in vitro, which is expected to exert an advantageous effect on bone defect repair and regeneration.

### In vivo bone repair

**Fig. 8:**
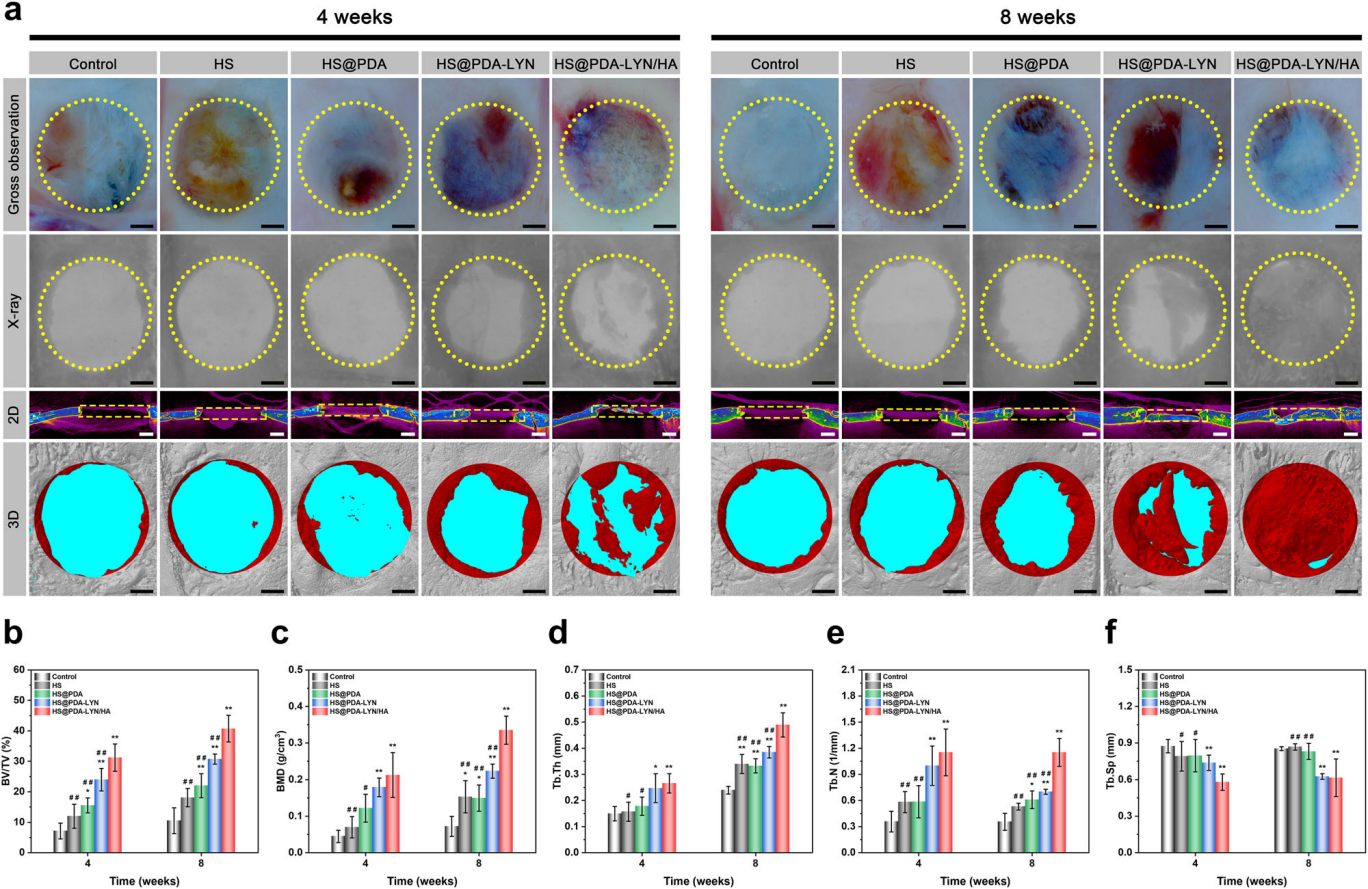
Radiological analysis of bone formation in vivo. **a** Representative macrophotographs, X-ray, and micro-CT images of the calvarial defect at 4 and 8 weeks after implantation. The yellow dotted lines indicate the initial boundary of critical-sized cranial defects. The red zones indicate the newly formed bone tissue in the defect areas. Summarized data of **b** BV/TV, **c** BMD, **d** Tb.Th, **e** Tb.N, and **f** Tb.Sp of the calvarial defect area. Scale bar in **a**: 1 mm. Data are expressed as the mean ± SD (*n* = 4). *P < 0.05 and **P < 0.01 indicate significant differences compared with the control group. ^#^*P* < 0.05 and ^# #^*P* < 0.01 indicate significant differences compared with the HS@PDA-LYN/HA group.

In this section, rat cranial bone defect models were established on thirty 8-week-old male SD rats to assess the bone repair efficacy and biocompatibility of fabricated scaffolds following established protocols^20,24^. The surgical procedures of material implantation are displayed in Supplementary Fig. 11. During the whole experimental period, animals that underwent surgery survived, and no signs of wound complications or infections occurred. At 4 and 8 weeks, the animals were sacrificed, and the in vivo bone repair efficacy was evaluated by macroscopic optical imaging, X-ray, micro-CT, histological and immunohistochemical studies. As shown in Fig. 8a, the gross morphological observations indicated that the residual material appeared obscure at the edges and integrated well with the surrounding tissues without displacement at 4 weeks after implantation. As the implantation time increased, the residual material became thin and completely bonded with the surrounding tissues, and the underlying space was partially filled with bony tissue at 8 weeks after implantation. Evidently, the newly developed scaffold was well accepted by the host bone without any harmful effect on the surrounding tissues. Subsequently, X-ray, two-dimensional (2D) micro-CT, and 3D micro-CT images of the regenerated bone were obtained, as shown in Fig. 8a. In accordance with the expected results, only a small amount of newly regenerated bone formed at the defect edges of the HS and control groups, and most of the defects remained unhealed, indicating that the defect (diameter: 5 mm) had an inability to self-heal, known as critical size. In contrast, various degrees of regenerated mineralized matrices were observed on the HS@PDA, HS@PDA-LYN, and HS@PDA-LYN/HA groups from the edge to the center of the defects as the implantation time was extended. Especially in the HS@PDA-LYN/HA scaffold group at 8 weeks, new bone tissue almost filled the entire defect area, followed by the HS@PDA-LYN and HS@PDA groups. In view of this phenomenon, we speculated that the 3D porous and interconnected structures of HS-based scaffolds could act as a template to assist bone progenitor cells in adhering and proliferating, which contributed to the formation of regenerated bone in the defect area. More importantly, the bone repair ability in the HS@PDA-LYN/HA scaffold group was superior to that in the other groups at each time point, possibly due to the therapeutic drugs and ions released from HS@PDA-LYN/HA, which can be deduced from the results of the in vitro studies. Another reason for this enhancement is bioactive HA, which plays a positive role in osteoinductivity and could provide sufficient mechanical and space support for cell growth, proliferation, and differentiation, thereby accelerating bone regeneration^56^. Based on the micro-CT images, morphometric analyses of the region of interests (ROIs) were performed to quantify the bone repair efficacy, including the percentage of bone volume to total volume (BV/TV), bone mineral density (BMD), trabecular number (Tb. N), trabecular thickness (Tb. Th), and trabecular separation (Tb. Sp) (Fig. 8b–f). Overall, HS@PDA-LYN/HA and HS@PDA-LYN had higher BV/TV, BMD, Tb.Th, and Tb.N compared to the HS@PDA, bare HS, and control groups. However, the Tb. Sp exhibited the reverse trend, with lower values detected in the HS@PDA-LYN/HA and HS@PDA-LYN groups. Four weeks postimplantation, the BV/TV (indicative of new bone volume) value of the HS@PDA-LYN/HA scaffold group was 31.2 ± 4.5%, which was significantly higher than that of the other groups (control group: 7.1 ± 2.6%, HS: 11.9 ± 3.9%, HS@PDA: 15.5 ± 2.5%, and HS@PDA-LYN 23.9 ± 3.7%). With an extended implantation time, the HS@PDA-LYN/HA group exhibited the maximum BV/TV (40.7% ± 4.4%) value at 8 weeks after surgery, which was significantly higher than that of the HS@PDA-LYN group (30.7 ± 1.6%); likewise, the BMD, Tb.Th, and Tb.N of the HS@PDA-LYN/HA scaffold group were significantly higher. These positive results further substantiated the micro-CT images, indicating that the formation and thickening of new bone tissue was effective in the HS@PDA-LYN/HA scaffold group.

**Fig. 9:**
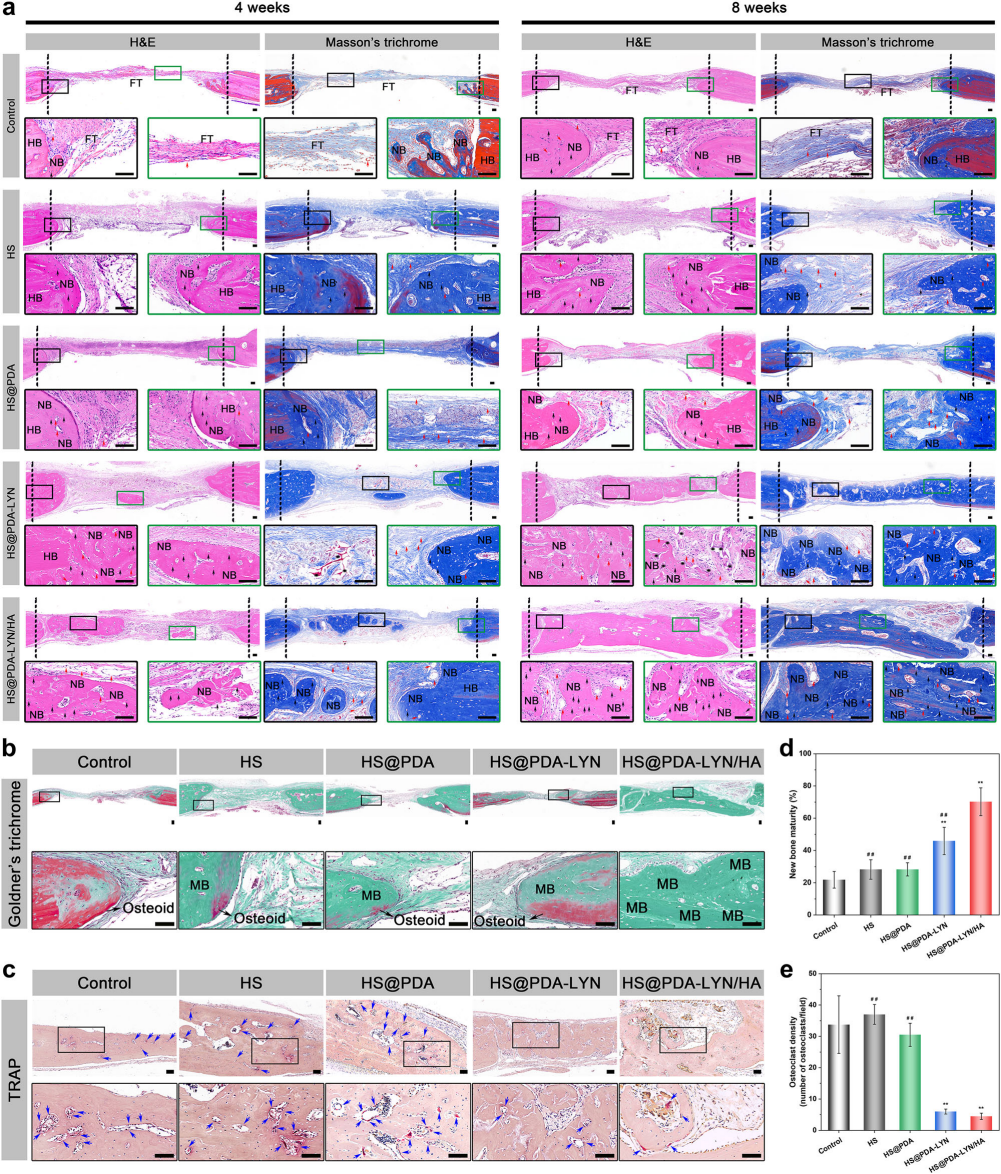
Histomorphological examination of the bone defect region after different treatments. **a** Representative H&E staining and Masson’s trichrome staining of the calvarial defect area at 4 and 8 weeks after implantation. Representative **b** Goldner’s trichrome staining and **c** TRAP staining of the calvarial defect area at 8 weeks postimplantation. Black arrows = osteoblasts; red arrows = blood vessels; blue arrows = osteoclasts; black asterisks: residual material; HB = host bone; NB = newly formed bone; FT = fibrous tissue; MB = mature/mineralized bone. Black dotted lines indicate the boundary of the rat calvarial defect. Quantitative analysis of **d** the percentage of newly formed mature bone area and **e** the number of osteoclasts in the regenerated tissues. Scale bar in **a**, **b**: 100 μm. Data are expressed as the mean ± SD (*n* = 4). *P < 0.05 and **P < 0.01 indicate significant differences compared with the control group. ^#^*P* < 0.05 and ^# #^*P* < 0.01 indicate significant differences compared with the HS@PDA-LYN/HA group.

After the micro-CT evaluation and morphometric analysis, histological analysis of regenerated new bone in defects was further assessed by hematoxylin and eosin (H&E) and Masson’s trichrome staining. As shown in Fig. 9a, H&E staining revealed that all scaffolds were degraded to certain levels at 4 and 8 weeks after the operation, and no obvious inflammatory response or necrosis was observed in the skull defects. Consistent with the X-ray and micro-CT results, much more new bone formation spreading from the margin to the center of the defect was observed in the HS@PDA-LYN and HS@PDA-LYN/HA scaffold groups, especially in the HS@PDA-LYN/HA scaffold group. In comparison, for the control, HS, and HS@PDA groups, the defect region was mainly filled with fibrous tissue, and only sparse bone tissue was found in the marginal defect region at 4 and 8 weeks after implantation. Notably, a high amount of bone matrix was observed in the HS@PDA-LYN/HA scaffold group, and the thickness was similar to that of the host bone, which implied significant bone regeneration at 8 weeks after implantation. Meanwhile, quantitative analysis of ossified tissue indicated that the HS@PDA-LYN/HA group exhibited the highest percentage of new bone area, followed by the HS@PDA-LYN group and the HS@PDA group, with the control group having the lowest percentage (Supplementary Fig. 12). Additionally, Masson’s trichrome staining was applied to detect newly formed bone tissue and blood vessels in the defect area. As shown in Fig. 9a, much denser blue- and red-stained osteoid islands and conspicuous neovascularization (labeled as red arrows) were found in the HS@PDA-LYN/HA scaffold group, which could accelerate bone regeneration due to the synergistic effect between vessels and bone. However, less osteoid formation was detected in the control, HS, and HS@PDA scaffold groups, which was consistent with the X-ray and micro-CT results. Moreover, both H&E and Masson’s trichrome staining revealed more newly formed osteoblasts (labeled as black arrows) in the HS@PDA-LYN/HA scaffold group, further evidencing the enhanced osteogenic potential. Furthermore, in vivo bone mineralization and remodeling were verified via Goldner’s trichrome staining (Fig. 9b) and TRAP staining (Fig. 9c), respectively. As expected, abundant immature bone (osteoid, stained orange/red) and osteoclasts (stained claret) were observed in the control, HS, and HS@PDA groups at 8 weeks after implantation, indicating poor bone repair quality in these groups. Conversely, a larger amount of mineralized bone in dark green together with few TRAP-positive osteoclasts was observed in the defect area of the HS@PDA-LYN and HS@PDA-LYN/HA groups, especially in the HS@PDA-LYN/HA group, implying elevated osteogenesis and decreased osteoclastogenesis. Quantitative analysis of the percentage of the newly formed mature bone area and the number of osteoclasts further substantiated the histological observations (Fig. 9d, e). Taken together, these data revealed that the HS@PDA-LYN/HA scaffold led to the dual capacity to promote osteogenesis and inhibit osteoclastogenesis in the defect area, which was related to the complementary and synergistic effects of LYN and HA, consistent with the in vitro biological performance results.

**Fig. 10:**
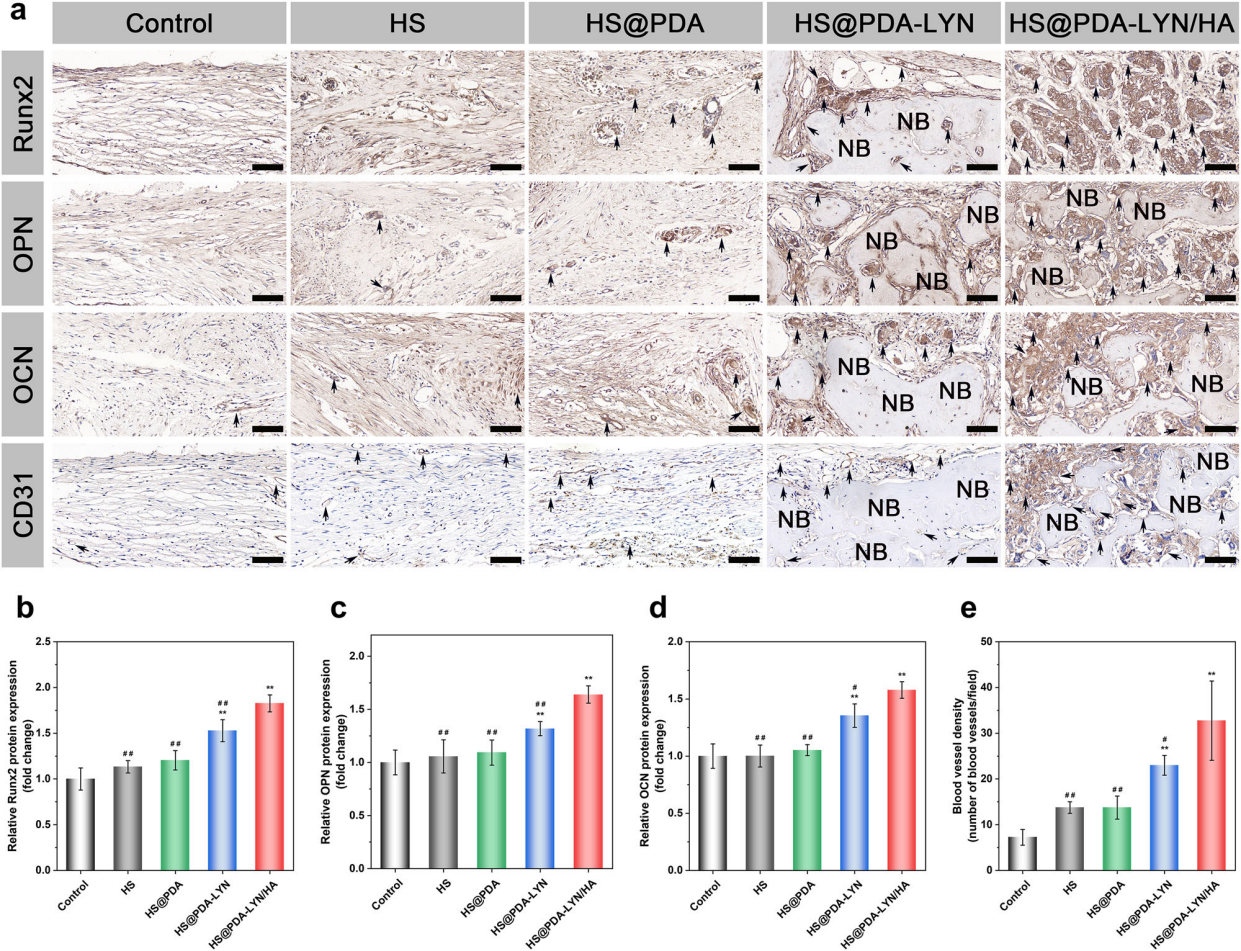
Immunohistochemical staining for osteogenesis and vascularization in the calvarial defect area. **a** Representative immunohistochemical staining of Runx2, OPN, OCN, and CD31 in newly formed tissues of the defect areas at 8 weeks postimplantation. The black arrows indicate the target protein-positive zone in the defect areas. NB = newly formed bone. Quantitative analysis of **b** Runx2, **c** OPN, **d** OCN, and **e** regenerated blood vessels. Scale bar in **a**: 100 μm. Data are expressed as the mean ± SD (*n* = 4). *P < 0.05 and **P < 0.01 indicate significant differences compared with the control group. ^#^*P* < 0.05 and ^# #^*P* < 0.01 indicate significant differences compared with the HS@PDA-LYN/HA group.

Decalcified bone tissue samples were further immunohistochemically stained to detect bone formation (Runx2, OPN, and OCN) and neovascularization (CD31) in the defect area at 8 weeks after implantation. Generally, Runx2 is an early osteogenic differentiation marker, whereas OPN and OCN are usually expressed at the middle/late stage of differentiation^57^. According to the immunohistochemical results (Fig. 10a–d), the HS@PDA-LYN/HA group had remarkably higher expression of Runx2, OPN, and OCN than the other groups at 8 weeks postimplantation, while the control and pure HS groups had almost no apparent positive staining. Thus, HS@PDA-LYN/HA significantly accelerated matrix mineralization and bone regeneration in the defect area, which was associated with the fact that HS@PDA-LYN/HA could stimulate both early and late osteogenic differentiation and provide a suitable microenvironment to favor bone formation. From the abovementioned results, it was deduced that our prepared HS@PDA-LYN/HA scaffold could support in situ bone regeneration by effectively promoting the differentiation of osteoblasts and the mineralization of bone matrix, exhibiting robust osteoinductive potential. Additionally, the timely construction of the vascular system also plays a predominant role in bone tissue regeneration, as the growth of new bone tissue depends on its internal blood supply and the surrounding capillaries. Therefore, vascular-promotive ability is an attractive property for BTE scaffolds, which is also a prerequisite for successful bone repair^10^. In this work, CD31 was selected as a specific marker of capillary endothelial cells to identify the distribution of blood vessels. As shown in Fig. 10a, the HS@PDA-LYN/HA group had significantly more CD31-positive vessels than the other groups, indicating the superior stimulation of vascularized bone regeneration by the HS@PDA-LYN/HA scaffolds. Correspondingly, quantitative analysis of the newly formed blood vessels by measuring the average number of vessels (Fig. 10e) and blood vessel area (Supplementary Fig. 13) based on immunohistochemical staining of CD31 further validated this observation. The enhanced angiogenic capacity of the HS@PDA-LYN/HA scaffold corroborated previously published studies^58^, in which increased expression of Runx2, OPN, and OCN was usually accompanied by the formation of new blood vessels.

Beyond these superior biological performances for bone regeneration, the in vivo biosafety of implanted biomaterials is also a critical factor that should be considered for practical application [43]. Therefore, we employed H&E staining to analyze the potential toxicity of all scaffolds in vivo. Supplementary Fig. 14 shows histological slices of organs collected from the sacrificed rats, including the heart, liver, spleen, lung, and kidney. The results indicated no apparent organ damage or abnormalities among all groups, suggesting that the implantation of the surface-modified HS 3D scaffold did not cause any side effects in terms of in vivo biosafety. Overall, the in vivo study results further confirm the biocompatibility of HS@PDA-LYN/HA and indicate favorable pro-osteogenic and anti-osteoclastic (primarily) and pro-angiogenic (additionally) activities of HS@PDA-LYN/HA, which can be considered a promising multifunctional material for accelerating bone regeneration.

In summary, inspired by mussel adhesive proteins, we successfully designed and constructed a surface-modified HS 3D scaffold with LYN and HA, which exhibited active anti-osteoclastic and osteogenic abilities in vitro and in vivo. Immobilized LYN was released from the scaffolds in a sustained manner and used to target osteoclast precursor cells to inhibit osteoclastogenesis in vitro. Furthermore, biomineralized HA nanocrystals were successfully precipitated on the surface of the porous 3D HS scaffold, which endowed scaffolds with potent osteogenic activity, providing a beneficial microenvironment for the attachment, proliferation, spreading, and differentiation of MC3T3-E1 cells and rBMSCs. In vivo evaluation further mirrored the aforementioned results and demonstrated that the 3D hybrid scaffold induced favorable osteogenesis and angiogenesis while deactivating osteoclastogenesis, ultimately resulting in robust bone regeneration. Based on these evaluations, we consider that the HS@PDA-LYN/HA scaffold would serve as a potential bone graft scheme for future clinical use in repairing large bone defects.

## Methods

### Materials

Hydroxyethyl cellulose (viscosity, 30,000 mPa) was obtained from Shandong Head Reagent Co. (Shandong, China). Soy protein isolate (Mw: 2.05 × 10^5^) was acquired from DuPont Protein Technology (Luohe, China). Epichlorohydrin (ECH), hydrochloric acid (HCl), and acetic acid were purchased from Sinopharm Chemical Reagent Co. (Shanghai, China). Reagent-grade chemicals, including dopamine hydrochloride (PDA, 98%), tris (hydroxymethyl) aminomethane (Tris), NaCl, KCl, CaCl_2_·2H_2_O, MgCl_2_·6H_2_O, NaH_2_PO_4_·H_2_O, and NaHCO_3_, were used to prepare the Si-containing precursor solution and purchased from Sigma-Aldrich Trading Co., Ltd., Shanghai, China. Mouse calvaria-derived MC3T3-E1 preosteoblastic cells and HUVECs were purchased from the Cell Bank of the Chinese Academy of Sciences (Shanghai, China). Fetal bovine serum (FBS), alpha-modified Eagle’s medium (α-MEM), Dulbecco’s modified Eagle’s medium (DMEM), phosphate-buffered saline (PBS), trypsin-EDTA, and penicillin/streptomycin (P/S) were obtained from Gibco (Gibco, Grand Island, NY) for cell culture in vitro. The CCK-8 assay was purchased from Dojindo Laboratories, Kumamoto, Japan. The live/dead cell staining kit was purchased from BestBio Biotechnologies (Shanghai, China). Triton X-100 (Sigma-Aldrich), DAPI (Sigma-Aldrich), and TRITC-labeled phalloidin (Invitrogen) were used for cell staining. The Annexin V-FITC apoptosis detection kit, RIPA lysis buffer, TRIzol RNA extract kit, 5-bromo-4-chloro-3-indolyl phosphate/nitro blue tetrazolium (BCIP/NBT) Alkaline Phosphatase Color Development Kit, and bicinchoninic acid (BCA) protein assay kit were purchased from Beyotime Biotechnology Co. (Jiangsu, China). Matrigel^®^ Matrix Growth Factor Reduced Basement Membrane was purchased from Corning (Corning Incorporated, Corning, NY, USA). The alkaline phosphatase assay kit was provided by Jiancheng Biotech Institute (Nanjing, China). All other reagents and solvents were purchased from Guangzhou Chemical Corporation and were of analytical grade without further purification. The water used in all experiments was purified by a Milli-Q cycle purification system (Millipore, USA).

### Fabrication of HS-based porous scaffolds

The ECH-crosslinked HS porous scaffold was fabricated via a combination of blending, crosslinking, and freeze-drying processes, as we have previously reported^26^. In brief, 2% (w/w) HEC and 10% (w/w) SPI precursor solutions were mixed thoroughly at a 3:7 volume ratio under continuous stirring at room temperature for 24 h. After sufficient crosslinking with HEC/SPI by adding ECH, the final hydrogels were transferred into a freezer at −20 °C overnight to form a randomly distributed crystallization network, followed by freeze-drying. Finally, the cross-linked scaffolds were washed with deionized (DI) water three times to remove the byproducts of the chemical reactions, followed by freeze-drying to obtain the porous structure of the scaffolds.

### PDA coating and immobilization of LYN

The as-prepared HS porous scaffolds were first immersed in a dopamine hydrochloride (10 mM Tris, pH = 8.5) solution (2 mg/mL) and incubated on a shaker at room temperature for 24 h. To ensure uniform deposition onto surfaces and prevent the formation of PDA aggregates in solution, the solution was replaced every two hours with fresh solution. After rinsing with PBS to remove the unreacted dopamine, the PDA-coated scaffold (HS@PDA) was frozen at −80 °C and freeze-dried for further applications. For LYN immobilization, PDA@HS was immersed in LYN solution (50 μg/mL in 10 mM Tris-HCl, pH 8.5) for 12 h at room temperature. Subsequently, the hybrid scaffolds (HS@PDA-LYN) were rinsed three times with 10 mM Tris-HCl (pH = 8.5) to remove unattached LYN and then freeze-dried to acquire the final HS@PDA-LYN 3D hybrid scaffolds.

### In situ biomineralization of HA

Simulated body fluid (10 × SBF) solutions were prepared according to the following protocols^59^. Briefly, 1000 × 10^−3^ M NaCl, 25 × 10^−3^ M CaCl_2_, 5 × 10^−3^ M MgCl_2_, 10 × 10^−3^ M NaH_2_PO_4_, and 10 × 10^−3^ M NaHCO_3_ were sequentially added to deionized water. Afterward, HS@PDA-LYN was immersed in 10 × SBF to induce HA nanocrystal deposition. The system was incubated at 37 °C for 24 h to accelerate mineral deposition on the scaffold surface. Ultimately, the mineralized scaffolds (HS@PDA-LYN/HA) were removed from the 10 × SBF solution, gently washed with DI water to remove unreacted substances, and subsequently lyophilized at −50 °C for 24 h under vacuum. Before further use, all samples were sterilized with ethylene oxide (EO) gas in an EO sterilizer (AN741, HW Anderson, USA) and maintained dry. The whole fabrication scheme of the 3D hybrid scaffolds is illustrated in Fig. 1a.

### Characterization of the hybrid scaffolds

Before testing the following characterization, the scaffold samples were rinsed with DI water to remove potential unchelated drugs and ions. The surface and cross-sectional morphologies of various scaffolds at different magnifications were observed by SEM (VEGA3, TESCAN, Czech Republic) with an accelerating voltage of 20 kV. The elemental analysis and chemical composition were characterized by EDS (Gemini 300, ZEISS, Germany) and XPS (ESCALAB 250XI, Thermo Scientific, New York). The 3D microstructure of various scaffolds was characterized by high-resolution micro-CT (SkyScan 1276, Bruker, Germany) with an image pixel size of 3 µm at 34 kV and 170 µA. The average pore size of various scaffolds was assessed by cross-sectional SEM images, calculated by measuring at least thirty pores using ImageJ software (NIH, Bethesda, MD). The porosity of various scaffolds was measured by the auxiliary histomorphometric software (CTAn) of micro-CT according to a published protocol described by Golafshan et al.^60^. The surface hydrophilicity of various scaffolds was analyzed by measuring the WCAs with the sessile drop shape method and a drop analysis system (DSA25; KRUSS, Hamburg, Germany). The crystal structure of various scaffolds was detected using an X-ray diffractometer (XRD-6000, Shimadzu) equipped with CuKα radiation (λ = 1.540598 Å). Data were collected over the 2θ region from 5° to 40° with a speed of 2°/min. The chemical functional groups of various scaffolds were determined using FTIR spectroscopy (TNZ1-5700, Nicolet, USA) with an attenuated total reflection (ATR) unit. The spectra were obtained in transmission mode over a wavenumber range of 4000–500 cm^−1^. Compression testing was performed by using a universal testing machine (CMT6503, Shenzhen SANS Test Machine, China) in aqueous medium. The measurement was performed at a compression extension speed of 1 mm/min for stress–strain tests with a load cell of 100 N at room temperature. The immobilization amounts of LYN and inorganic components were evaluated using thermal gravimetric analysis (TGA, Diamond TG/DTA; PerkinElmer Instruments, Shanghai, China). The samples were submitted to TGA measurement in a nitrogen atmosphere from 25 to 800 °C at a heating rate of 10 °C/min. The in vitro release profile of LYN and Ca ions from the hybrid scaffold was performed as previously described^61^. In brief, the lyophilized scaffold samples were soaked in 10 mL PBS solution (pH = 7.4) under shaking (70 rpm) at 37 ± 1 °C. At different predetermined time points (days 1, 3, 7, 14, 21, and 28), 8 mL of supernatant was collected from the incubation medium that was then replenished with the same volume of fresh PBS. To determine the concentration of the released Ca ion, the liquid (4 mL) was submitted for analysis by inductively coupled plasma atomic emission spectroscopy (ICP–AES, Varian 715 ES, California, USA). To determine the amount of released LYN, another 4 mL of liquid was measured by HPLC–MS/MS (TSQ Quantiva triple quadrupole mass spectrometer (Thermo Scientific) coupled to an UltiMate 3000 XRS HPLC system (Dionex, Thermo Scientific)

### Cell acquisition and culture

In vitro studies of osteogenesis and osteoclastogenesis were performed with MC3T3-E1 preosteoblastic cells and BMMs, respectively. BMMs were isolated from 4-week-old wild-type C57/BL6 male mice according to previously reported procedures^44,62^. All animal protocols were approved by the Animal Care and Use Committee of Wuhan University. In brief, the femur and tibia bones were separated aseptically, and both ends were cut and washed with α-MEM to obtain bone marrow cells. After removing red blood cells, the residual marrow cells were cultured in complete medium comprising α-MEM supplemented with 10% FBS and 1% P/S and incubated in a 5% CO_2_ atmosphere at 37 °C. After 24 h of incubation, suspended cells were collected and cultured in α-MEM complete medium supplemented with 30 ng/mL macrophage-stimulating factor (M-CSF). To identify BMMs, FITC-labeled anti-CD11b antibody was employed through flow cytometry (FC500, Beckman Coulter, Fullerton, CA, USA) as previously described^63^. Flow cytometry data were assessed using FlowJo software (version 10.7.1) and used the same gating strategies. A supplementary figure to graphically account for FACS sequential gating strategies has been provided (Supplementary Fig. 3). MC3T3-E1 cells and HUVECs were maintained in DMEM containing 10% FBS and 1% P/S under 5% CO_2_ and a humidified atmosphere of 37 °C. In addition to MC3T3-E1 cells, rBMSCs were further isolated to assess cell adhesion and osteogenic differentiation according to our established protocol^31^. Cells were passaged using 0.25% trypsin-EDTA when they reached 80–90% confluency. The culture medium was changed every two days. All cell handling procedures were performed in a sterile laminar flow hood.

### In vitro evaluation of cytocompatibility

The in vitro cytocompatibility of the scaffolds was evaluated by both extraction and direct contact methods, as previously reported^64^. The scaffold extracts were prepared based on the international standard ISO 10993-12: 2012. Referring to our previous work^27^, sterilized scaffold samples were incubated in α-MEM or DMEM (0.2 g of powder per 1 mL culture medium) containing 1% (v/v) P/S at 37 °C with a shaking speed of 120 rpm. After 72 h of extraction, the supernatant was collected by centrifugation at 1000 rpm for 5 min and then filtered through a 0.22 μm filter (PALL, USA). Acquired extracts of each sample were supplemented with 10% FBS for the following in vitro cell experiments.

The proliferation and viability of MC3T3-E1 cells and BMMs were detected by a CCK-8 assay based on the manufacturer’s instructions. In brief, MC3T3-E1 cells and BMMs were seeded in 96-well plates at a density of 2 × 10^3^ cells per well. After 1 day of initial cell attachment, the growth medium was discarded and then replaced with sample extracts. Complete culture medium without sample extracts was used as a negative control. At the scheduled time points (1, 3, 5, and 7 days), the incubation medium was thoroughly removed, and CCK-8 reagent was added. After reaction with a mixed working solution for 2.5 h, the absorbance values of each well were measured at a wavelength of 450 nm with a plate reader (Multiskanfc, Thermo Scientific). Cell viability was calculated using the following formula: cell viability (%) = OD_Scaffold_/OD_control_ × 100%, where OD_Scaffold_ and OD_control_ represent the absorbance values of scaffold extract-treated groups and the control groups, respectively. For the cell apoptosis assay, cells at a density of 2 × 10^5^ cells/well were seeded in six-well plates and incubated with different scaffold extracts. After culturing for 3 days, cell apoptosis was detected by flow cytometry using an Annexin V-FITC apoptosis detection kit according to the manufacturer’s instructions. Annexin V-FITC was used to identify early apoptotic cells by showing green fluorescence, and PI was used to identify late apoptotic cells by showing red fluorescence. The following panels were generated for optimal results: forward scatter-height (FSC-H) versus side scatter-height (SSC-H) were used to capture all cells and exclude cell fragments from quantitative analysis (Supplementary Fig. 15). To further determine the cytocompatibility of the fabricated scaffolds, cells (1 × 10^5^ cells/mL) were cultured on the scaffolds for 3 days. After that, live/dead staining was performed according to our established protocol^31^. The fluorescent images were observed using CLSM (TCS SP8, Leica, Germany) with 488 nm (green, calcein-AM) and 561 nm (red, EthD-1) excitation filters. For z-series 3D images, the scanning height was 80 μm, and each step was 1 μm. Meanwhile, the metabolic activity of cells (5 × 10^4^ cells/mL) cultured on fabricated scaffolds was measured with a CCK-8 kit according to previously described protocols^65^.

To examine cell adhesion and morphology on different samples, we performed F-actin staining as previously described^39^. Briefly, after MC3T3-E1 cells (1 × 10^4^ cells/mL) were cultured on fabricated scaffolds for 7 days, the samples were fixed with 4% paraformaldehyde for 15 min followed by permeabilization with 0.1% Triton X-100 for 5 min. After rinsing with PBS, the samples were incubated with TRITC-labeled phalloidin for 60 min and counterstained with DAPI for 5 min at room temperature. The samples were washed several times with PBS and then photographed by CLSM with 405 nm (blue, DAPI) and 561 nm (red, phalloidin) excitation filters. Furthermore, we also observed the adhesion of cells on the scaffolds on day 7 by SEM. In brief, the cell/scaffold constructs were fixed with 2.5% glutaraldehyde for 8 h. After rinsing with PBS, the samples were dehydrated through a graded ethanol series (50, 70, 80, 90, and 100%) for 15 min each step. Then, they were vacuum-dried, sputter-coated with gold, and processed for SEM observation.

### In vitro evaluation of osteogenic differentiation

For osteogenic differentiation, the cells were seeded into 24-well culture plates at a density of 4 × 10^4^ cells per well. After incubation for 24 h, the culture medium was replaced by osteogenic induction medium (OIM: scaffold extracts supplemented with 10 mmol/L β-glycerophosphate disodium salt, 50 μg/mL ascorbic acid, and 10 nmol/L dexamethasone). In addition, the cells incubated in normal OIM (without scaffold extracts) containing the aforementioned osteoinductive factors served as a negative control. The medium was refreshed every two days.

### ALP activity assay

To investigate the early osteogenic differentiation potential, an ALP activity assay was performed as previously described^29^. After 7 days of induction, the samples were washed twice with PBS and fixed in 4% paraformaldehyde for 30 min. Subsequently, the fixed samples were stained with BCIP/NBT at room temperature for 2 h and finally observed using a light microscope (Olympus IX73, Tokyo, Japan). For the semiquantitative analysis of ALP activity, an ALP assay kit was utilized according to an established protocol. The total protein content of each sample was determined for normalization.

### ARS and Von Kossa staining assays

To investigate the calcium nodule formation and matrix mineralization abilities of various scaffolds, ARS staining was carried out according to a published protocol^29^. After 21 days of induction, the samples were washed twice with PBS and fixed in 4% paraformaldehyde for 30 min. Subsequently, the fixed samples were stained with 2% ARS solution for 15 min at room temperature. After rinsing with PBS, the samples were observed by inverted light microscopy. To quantify ECM mineralization, the cells stained with ARS were reacted with 10% cetylpyridinium chloride (Sigma-Aldrich) for 1 h at room temperature with shaking, and the absorbance values of each well were measured at a wavelength of 562 nm using a UV–Vis spectrophotometer (Perkin Elmer, USA). For Von Kossa staining, cells were incubated following the same process as mentioned above. After 30 days of induction, the samples were washed twice with PBS and fixed in 4% paraformaldehyde for 30 min. After that, the cells were soaked in 5% silver nitrate and then exposed to UV light for 10 min. Images were acquired by a light microscope. The quantification of the positive areas of Von Kossa-stained nodules was calculated using ImageJ software.

### Osteogenic gene and protein expression assays

Then, the expression levels of osteogenesis-related genes, including Runx2, Col-1, and OPN, were examined using qRT–PCR assays after 7 days of induction. Total RNA from the MC3T3-E1 cell lysates was extracted by adding TRIzol reagent. The concentration of purified total RNA was then determined using a NanoDrop spectrophotometer (Thermo Fisher Scientific, Wilmington, DE). One microgram of total RNA was reverse-transcribed to complementary DNA (cDNA) using the HiScript III RT SuperMix reverse transcription kit following the manufacturer’s protocol. After reverse transcription, qRT–PCR was performed in a 7500 Real-Time PCR system (Applied Biosystems, USA) with ChamQ SYBR qPCR Master Mix. The expression level of the target gene was normalized to the housekeeping gene glyceraldehyde-3-phosphate dehydrogenase (GAPDH). The primer sequences are displayed in Supplementary Table 1. The relative gene expression to the control group was calculated with the formula 2^−△△CT^. In addition, after culturing for 7 days, the expression of osteogenesis-related proteins (Runx-2, Col-1, and OPN) was detected by immunofluorescence staining as previously described^66^. Briefly, MC3T3-E1 cells were fixed in 4% paraformaldehyde and permeabilized with 0.1% Triton X-100 for 15 min to increase cell permeability. After rinsing with PBS, the samples were treated with 5% goat serum for 60 min to block nonspecific binding, followed by incubation with primary antibodies at a dilution of 1:100 overnight at 4 °C. Primary antibodies, including anti-Runx2 (Cell Signaling, D1L7F), anti-Col-1 (Affinity, AF7001), and anti-OPN (ProteinTech, 22952-1-AP), were used in this study. Subsequently, the samples were washed twice in PBS and incubated with Alexa Fluor^®^ 647-conjugated goat anti-rabbit IgG (ab150079, Abcam) at a dilution of 1:200 in the dark for 1 h at room temperature. After staining F-actin with phalloidin and nuclei with DAPI, the samples were imaged by CLSM.

### In vitro evaluation of osteoclastic differentiation

For osteoclastic differentiation, BMMs were seeded into 48-well culture plates at a density of 5 × 10^4^ cells per well. After 24 h of incubation, the culture medium was replaced by scaffold extracts supplemented with 50 ng/mL receptor activator of nuclear factor-κ B Ligand (RANKL) and 30 ng/mL M-CSF. The medium was refreshed every two days.

### TRAP and F-actin ring staining assays

After incubation with complete extract supplemented with osteoclast differentiation factors for 6 days, TRAP staining was used to evaluate the activity of osteoclasts in each group as previously described^62^. Briefly, cells were fixed with 4% paraformaldehyde for 30 min at room temperature and stained with TRAP solution (Servicebio, G1050-50T) for 30 min at 37 °C. To stop the extra staining, the cells were washed with DI water and further imaged with a light microscope. TRAP-positive multinucleated cells containing over three nuclei were considered osteoclasts. The number and area of TRAP-positive cells were quantified by using ImageJ software.

To further observe the effect of different scaffold extracts on the osteoclastogenesis of BMMs, immunofluorescence staining of F-actin rings was performed with the same method mentioned above. Briefly, BMMs were cultured with extracts containing RANKL and M-CSF for 6 days following the same process as aforementioned. After incubation with TRITC-labeled phalloidin and DAPI to counterstain the cytoskeleton and nuclei, respectively, immunofluorescence images were captured via an inverted fluorescence microscope and analyzed using ImageJ software to evaluate the number and area of the formed F-actin rings.

### Osteoclastic gene and protein expression assays

After culturing for 3 days, the expression of osteoclastogenesis-related genes and proteins, including NFATc1, CTSK, and RANK, was detected by qRT–PCR and immunofluorescence staining assays as mentioned earlier. The housekeeping gene GAPDH was used for normalization. The relative gene expression was calculated with the formula 2^−△△CT^ formula and represented as a fold change relative to the control. The primer sequences are displayed in Supplementary Table 1. To visualize osteoclastogenesis-related proteins in cells, immunocytochemistry was performed with primary antibodies at a dilution of 1:200 overnight at 4 °C. Primary antibodies, including anti-NFATc1 (Affinity, DF6446), anti-CTSK (Santa Cruz, sc-48353), and anti-RANK (Abcam, ab13918), were used in this study. Subsequently, the samples were incubated with Alexa Fluor® 647-conjugated goat anti-rabbit IgG (Abcam, ab150079) at a dilution of 1:200 in the dark for 1 h at room temperature. After staining the nuclei with DAPI, the samples were imaged by CLSM.

### Detection of the expression level of ULK1 in the osteoclastic differentiation of BMMs

For the qRT–PCR assay, the total RNA extraction process and qRT–PCR procedure were performed with the same method mentioned above. The final results are presented as the fold change relative to the control group. The primer sequences used are also listed in Supplementary Table 1. Furthermore, the protein expression levels were detected by western blot analysis of BMMs after 3 days of culture. Briefly, total cell proteins were extracted by immunoprecipitation lysis (Servicebio, Cat # G2038) containing protease inhibitor cocktail (MCE, Cat# HY-K0010, 1:100) and phosphatase inhibitor cocktail I (MCE, Cat# HY-K0021, 1:100). Protein (approximately 30 μg) was loaded in sodium dodecyl sulfate–polyacrylamide gel electrophoresis (SDS–PAGE) and then transferred to polyvinylidene difluoride (PVDF) membranes. Subsequently, the PVDF membranes were blocked with 5% (w/v) skim milk (BD Biosciences, Sparks, MD, USA) in TBST solution at room temperature for 120 min and then incubated with primary antibodies against ULK1 (#8054, CST, USA) at 4 °C overnight. After washing with TBST three times, the membranes were further incubated with secondary antibodies at room temperature for 60 min. GAPDH (Proteintech Group In, Cat# 60004-1-Ig, 1:4000) was used as the reference gene. The target bands were visualized by a Tanon-5200 chemiluminescent imaging system (Tanon, Shanghai, Beijing).

### In vitro evaluation of angiogenesis

To evaluate the effect of different scaffolds on the angiogenic ability of HUVECs in vitro, phalloidin/DAPI staining, and tube formation assays were performed according to a previously described protocol^67^. In brief, after HUVECs (1 × 10^5^ cells/mL) were cultured on fabricated scaffolds for 3 days, the cell-laden scaffolds were fixed, followed by incubation with TRITC-labeled phalloidin for 60 min at room temperature. The cellular nuclei were counterstained with DAPI. Finally, fluorescent images were captured using CLSM. For tube formation assays, HUVECs at a density of 5 × 10^4^ cells/well were seeded in 24-well plates precoated with growth factor-reduced basement membrane matrix (Matrigel) and incubated with scaffold extracts from different groups. Cells cultured with endothelial cell medium alone were used as a negative control. After incubation for 8 h, the morphological changes of the cells and the tube-like structures were photographed using a light microscope. Tube formation, including the number of branches and junctions, was carefully quantified using ImageJ software with the Angiogenesis Analyzer plugin (NIH, Bethesda, MD, USA). Furthermore, the angiogenic properties of HUVECs were evaluated by immunofluorescent staining of CD31 following the manufacturer’s instructions. Briefly, after incubating for 7 days, cells were fixed and incubated with primary anti-CD31 antibody (H-3; PECAM-1. Santa Cruz, SC-37676) overnight at 4 °C. After that, the cellular actin and nuclei were counterstained with FITC-phalloidin and DAPI, respectively. Finally, the fluorescent images were observed using CLSM.

### Surgical procedure

All animal procedures were performed in line with the Animal Ethical Committee of Wuhan University, and the methods in the current work were performed in compliance with “Guiding Opinions on the Treatment of Animals (09/30/2006)” published by the Ministry of Science and Technology of the People’s Republic of China.

In this study, a critical-sized calvarial defect model was established in Sprague–Dawley rats (*n* = 30, 8 weeks old, male) to evaluate bone regeneration efficacy. Briefly, all rats were randomly divided into five groups according to different repair materials as follows: (1) control group (bone defects without material, *n* = 6), (2) HS group (HS implantation, *n* = 6), (3) HS@PDA (HS@PDA implantation, *n* = 6), (4) HS@PDA-LYN (HS@PDA-LYN implantation, *n* = 6), and (5) HS@PDA-LYN/HA (HS@PDA-LYN/HA implantation, *n* = 6). After anesthetization with isoflurane, the rats were operated on in the prone position. The scalp area covering the calvarial vault was shaved and then prepped with an antiseptic solution (chlorhexidine) scrub. Subsequently, the skin, subcutaneous tissue, and periosteum were sequentially incised through a sagittal incision (~1.5 cm in length), leading to exposure of the calvarial bone. For each rat, bilateral full-thickness critical-sized calvarial defects (5 mm in diameter) were created on both sides along the midline using an electrical bone trephine bur. Meanwhile, saline solution was continuously added to reduce the temperature during drilling. The defects were then implanted with sterilized scaffolds (diameter, 5 mm; height, 1 mm), and the incision was carefully closed layer-by-layer with 4-0 nylon sutures. All procedures were performed by the same surgeons for all groups. For three consecutive days, rats undergoing surgery received 1 mg/kg meloxicam and 10 mg/kg enrofloxacin postoperatively. At 4 and 8 weeks after implantation, the rats were sacrificed by injection with an overdose of anesthetic, and the calvarial tissues were immediately harvested for further radiographic and histological assessments.

### Micro-CT analysis

After fixing in 4% paraformaldehyde for 24 h, the calvarial samples were scanned using a micro-CT imaging system (SkyScan 1276 system, Bruker, Germany) according to our established protocol^30^. 2D images of each specimen were obtained under 53 kV and 200 μA, with an image pixel size of 6.5 μm. After scanning, NRecon software was used to reconstruct the 3D structure of the samples, and the parameters were set as follows: smoothing = 4, ring artifact correction = 12, and beam hardening correction = 46%. To quantitatively evaluate new bone formation in the defect area, the ROI with a 5 mm diameter was selected to analyze the following parameters: percentage of BV/TV, BMD, Tb. N, Tb. Th, and Tb. Sp. The threshold of the binary image was set to 90, and the BMD of the new bone was corrected using rat BMD standard specimens.

### Histological analysis

Following the micro-CT scan, all fixed rat samples were decalcified with 10% EDTA (pH = 7.4) for 4 weeks at 37 °C, and the EDTA solution was changed every three days. After decalcification, the samples were dehydrated in a graded ethanol series (70–100%), cleared with xylene, and finally embedded in paraffin. After that, consecutive 5 μm-thick sections were obtained from the defect area and stained with H&E and Masson’s trichrome to evaluate bone formation and residual materials. Additionally, some deparaffinized sections were subjected to Goldner’s trichrome staining and TRAP staining to evaluate bone mineralization and remodeling. Furthermore, immunohistochemical staining was carried out following previously described procedures^57^. Briefly, the deparaffinized sections were first blocked with 5% bovine serum albumin (BSA) solution, followed by incubation with primary antibodies against Runx2, Col-1, and OPN for osteogenesis markers and CD31 for angiogenesis markers at a 1:100 dilution overnight at 4 °C. To assess the potential toxicity of implanted scaffolds in vivo, major organs, including the heart, liver, spleen, lung, and kidney, of the rats were collected and stained with H&E at 8 weeks. All the samples were imaged with an optical microscope and semiquantified by ImageJ software.

### Statistical analysis

The results were obtained from at least three independent experiments. All data collected in this work are expressed as the mean ± standard deviation (SD) and were analyzed with Origin 2018 software (Origin Lab Corporation, USA). Differences among groups were evaluated using one-way analysis of variance (ANOVA) followed by Tukey post hoc tests. In all evaluations, differences were considered significant at * or ^#^, represented by a *p* value < 0.05, and highly significant at ** or ^##^, with a *p* value < 0.01.

### Reporting summary

Further information on research design is available in the Nature Research Reporting Summary linked to this article.

## Supplementary information

Supplementary Information

REPORTING SUMMARY

## Data Availability

The data supporting this article are found within the text and the supplementary information file. Any additional data and the data that support the plots within this paper are available from the corresponding author upon reasonable request.
