# Supplementary material for: Mussel-inspired multifunctional surface through promoting osteogenesis and inhibiting osteoclastogenesis to facilitate bone regeneration
Source: NPJ Regen Med. 2022 May 13;7:29. doi: 10.1038/s41536-022-00224-9 (PMC9106696; doi:10.1038/s41536-022-00224-9)
Supplement: Supplementary file 1 — Supplementary Information [file 41536_2022_224_MOESM1_ESM.pdf]

Supplementary Information

Mussel-inspired multifunctional surface through promoting osteogenesis and inhibiting osteoclastogenesis to facilitate bone regeneration

Minhao Wu[#a], Yufeng Zhang[#a], Ping Wu[#b], Feixiang Chen[c], Zhiqiang Yang[a], Sheng Zhang[a], Lingfei Xiao[a], Lin Cai[a], Chong Zhang[a], Yun Chen[*c], Zhouming Deng[*a]

[a] Department of Spine Surgery and Musculoskeletal Tumor, Zhongnan Hospital of Wuhan University, 168 Donghu Street, Wuchang District, Wuhan 430071 Hubei, People's Republic of China

[b] College of Life Science and Technology Huazhong University of Science and Technology Wuhan 430074, China

[c] Department of Biomedical Engineering and Hubei Province Key Laboratory of Allergy and Immune Related Diseases, School of Basic Medical Sciences, Wuhan University, Wuhan 430071, China

# These authors contributed equally to this work.

* Correspondence should be addressed as follows:

Dr. Zhouming Deng. Email: dengzhouming@whu.edu.cn

Department of Spine Surgery and Musculoskeletal Tumor, Zhongnan Hospital of Wuhan University, 168 Donghu Street, Wuchang District, Wuhan 430071 Hubei, People's Republic of China

Prof. Yun Chen. Email: yunchen@whu.edu.cn

Department of Biomedical Engineering and Hubei Province Key Laboratory of Allergy and Immune Related Diseases, School of Basic Medical Sciences, Wuhan University, Wuhan 430071, China

This PDF file includes the following:

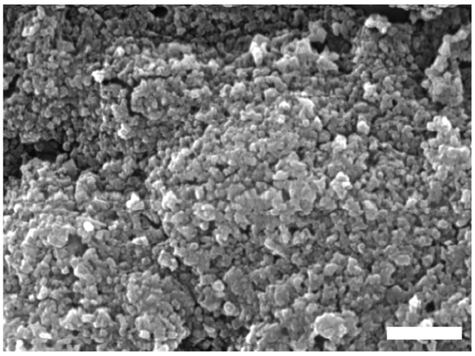

Supplementary Figure 1. Representative SEM images showing the in situ precipitation of HA nanocrystals, which were uniformly distributed on the surface of HS@PDA-LYN/HA. Scale bar: 2 μm.

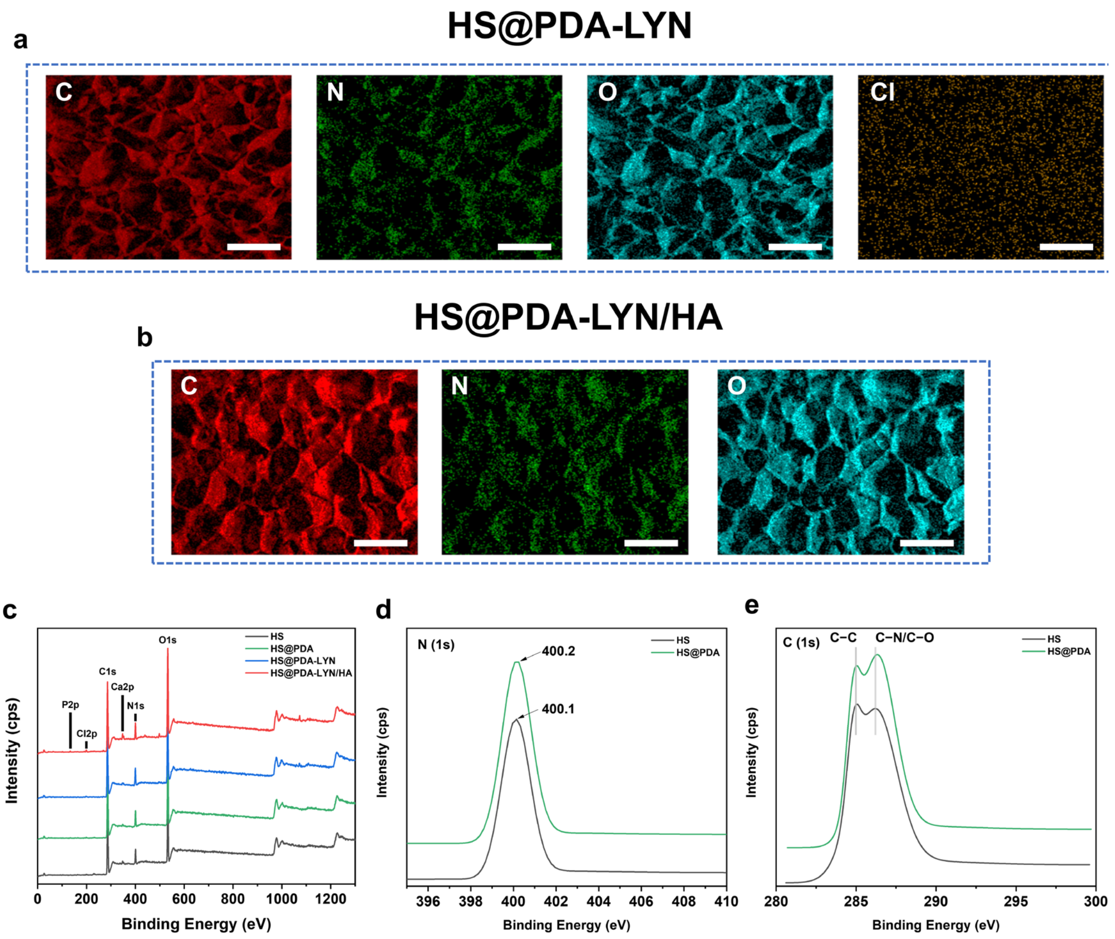

Supplementary Figure 2. EDS elemental maps of (**a**) HS@PDA-LYN and (**b**) HS@PDA-LYN/HA. (**c**) XPS analysis of the HS samples with different biofunctionalizations. High-resolution XPS spectra of (**d**) N 1s and (**e**) C 1s elements of HS and HS@PDA. Scale bar in a, b: 250 μm.

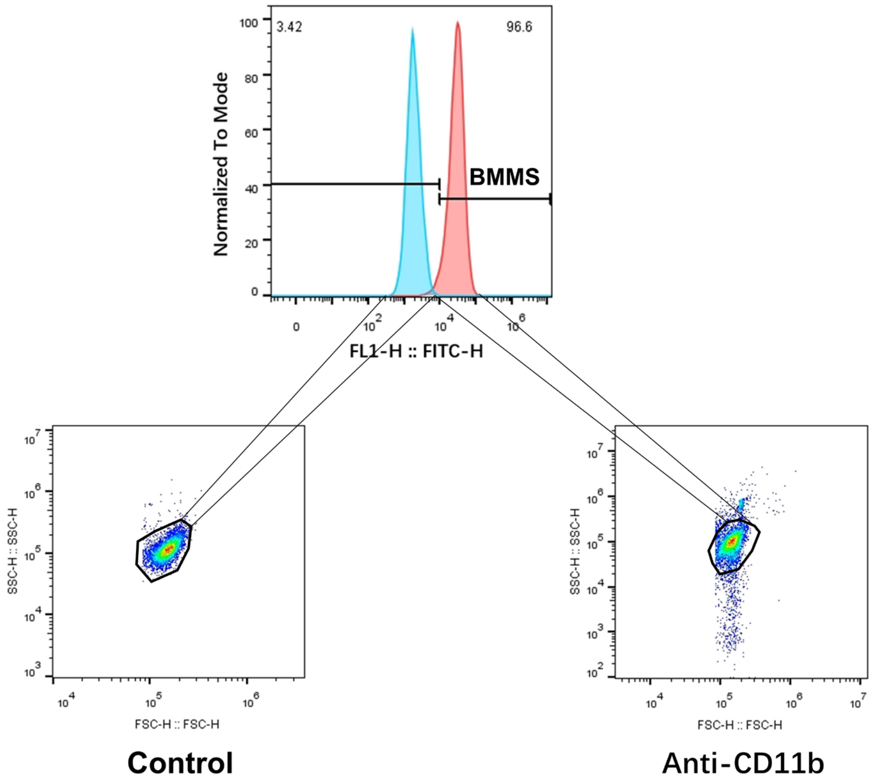

Supplementary Figure 3. Identification of BMMs (CD11b) analyzed by flow cytometry. FSC-H, forward scatter-height and SSC-H, side scatter-height.

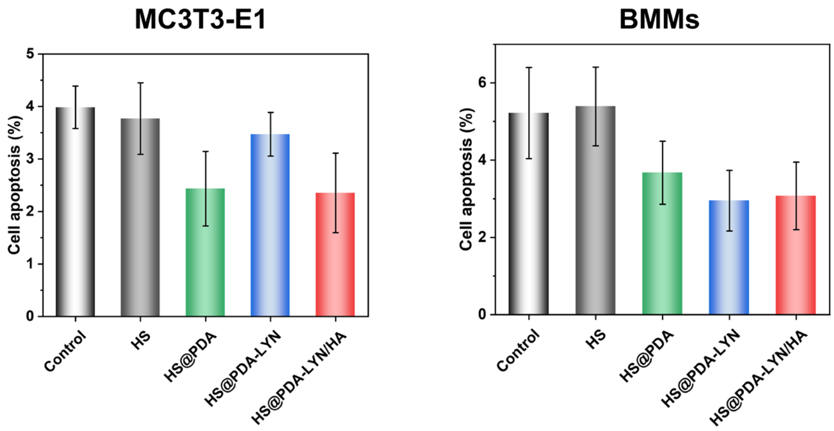

Supplementary Figure 4. Quantitative analysis of the cell apoptosis rate based on flow cytometry results. Data are expressed as the mean ± SD (n = 3).

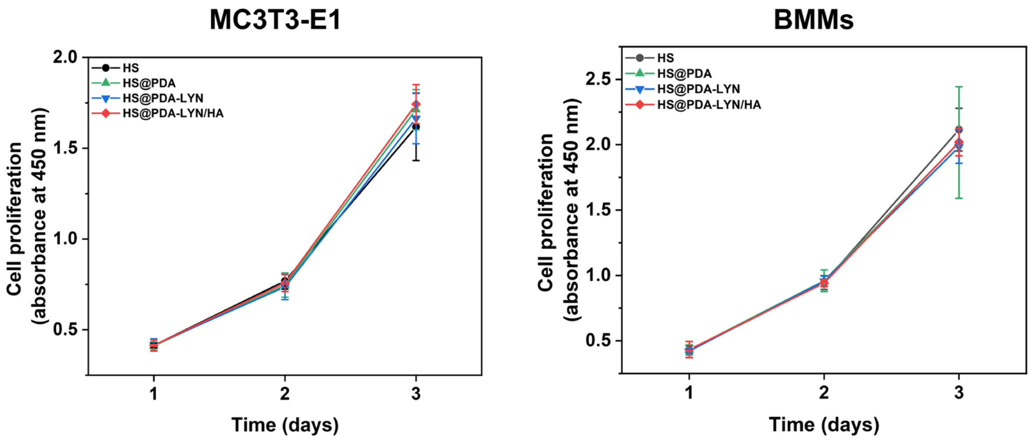

Supplementary Figure 5. The proliferation of MC3T3-E1 cells and BMMs cultured on different scaffolds for 1, 2, and 3 days. Data are expressed as the mean ± SD (n = 3).

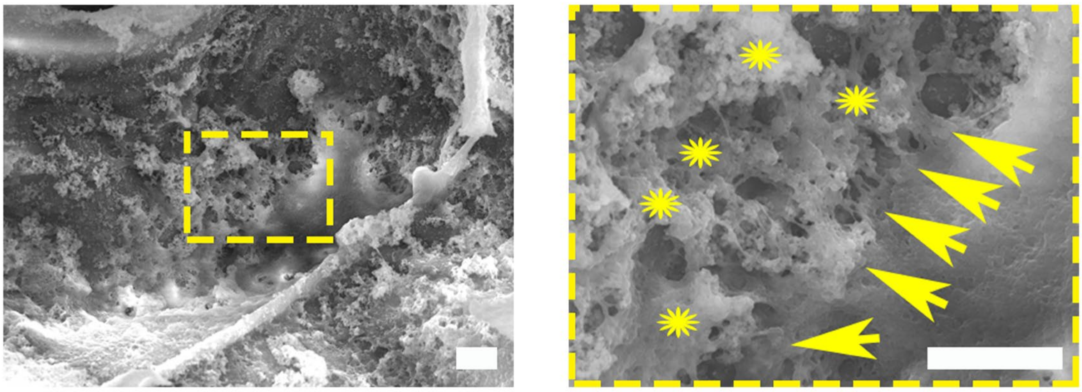

Supplementary Figure 6. Representative SEM images of MC3T3-E1 cells grown on HS@PDA-LYN/HA for 7 days. The yellow arrows indicate widespread filopodia and lamellipodia. The yellow asterisks indicate the strong interactions of the filopodia with the precipitated HA nanocrystals on the scaffold surface. Scale bar: 10 μm.

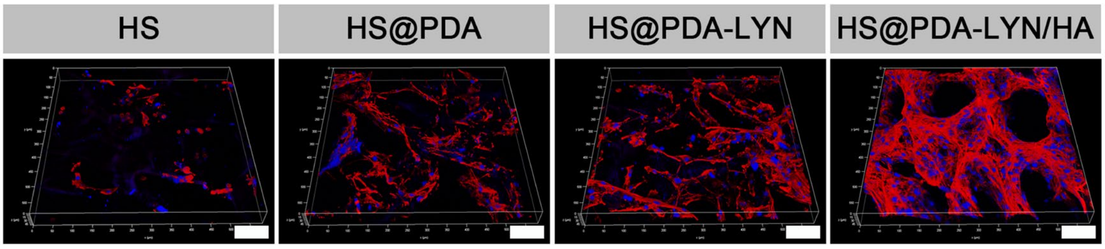

Supplementary Figure 7. Representative confocal Z-stack images of MC3T3-E1 cells cultured on different scaffolds after 7 days. F-actin and cell nuclei were labeled with fluorescent red and blue, respectively. Scale bar: 100 μm.

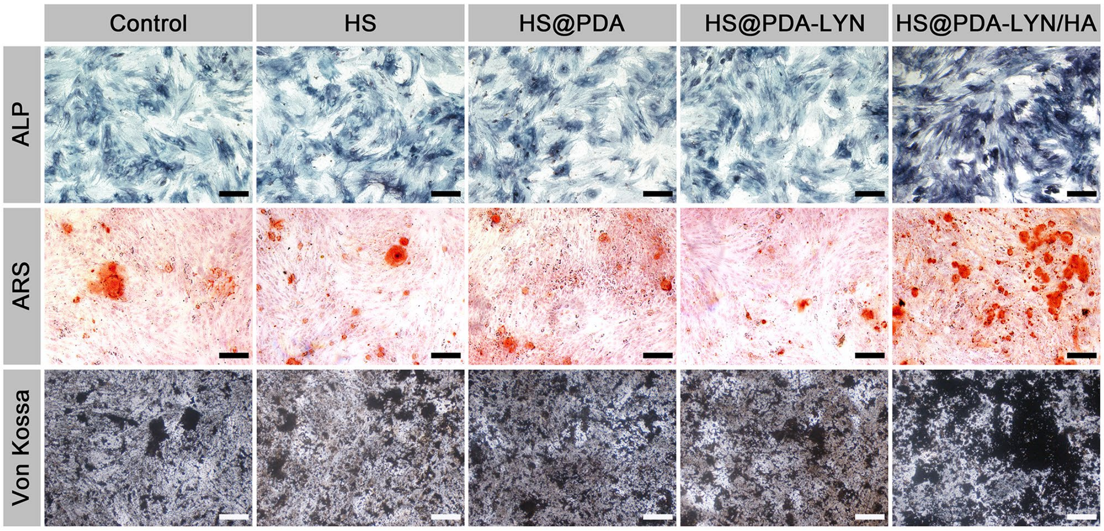

Supplementary Figure 8. Representative ALP staining, ARS staining and Von Kossa staining assays for rBMSCs incubated with different scaffold extracts for 7, 14, and 21 days. Scale bar: 200 μm.

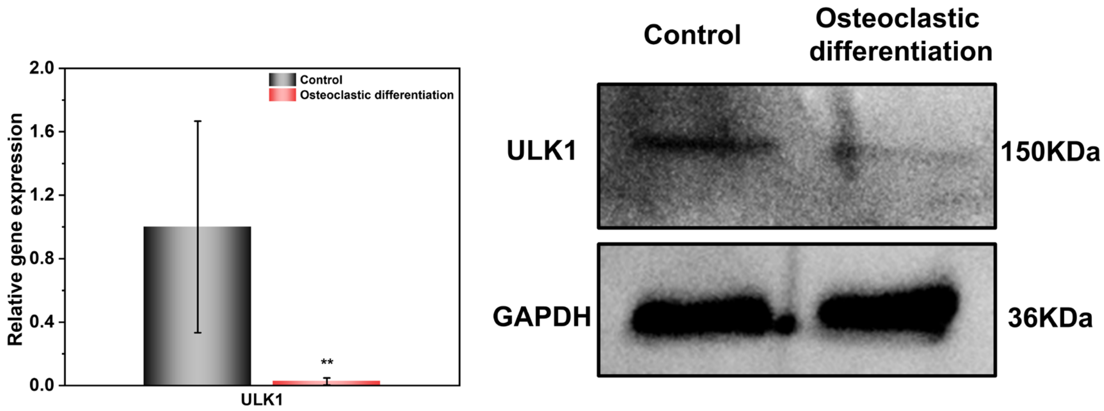

Supplementary Figure 9. (a) Quantitative analysis of relative ULK1 mRNA expression in BMMs during the osteoclastic differentiation process. **(b)** Representative western blot images showing the expression level of ULK1 in BMMs during the osteoclastic differentiation process. Data are expressed as the mean ± SD (n = 3). *P < 0.05 and **P < 0.01 indicate significant differences compared with the control group.

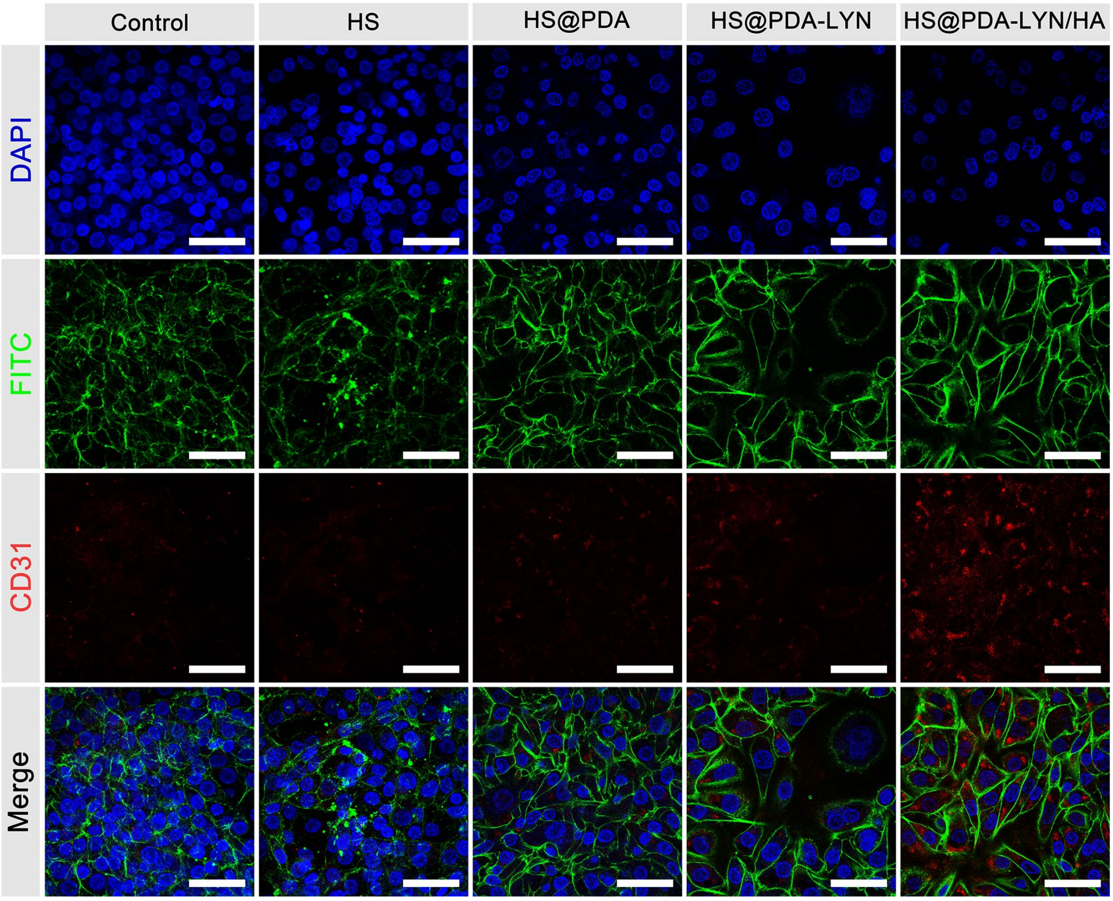

Supplementary Figure 10. Immunofluorescent staining of CD31 (red) in HUVECs incubated with different scaffold extracts for 7 days. F-actin and cell nuclei were labeled with fluorescent green and blue, respectively. Images were captured using confocal microscopy. HUVECs in the HS@PDA-LYN/HA group demonstrated the most abundant CD31 expression. Scale bar: 50 μm.

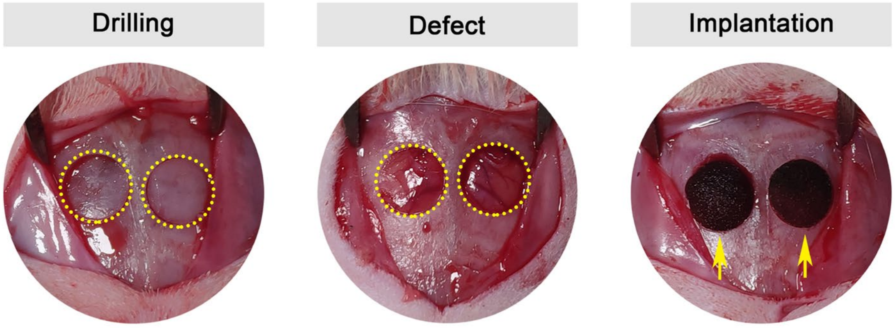

Supplementary Figure 11. Photographs of the surgical procedure during scaffold implantation. The yellow dotted lines indicate the boundary of the defect (5 mm in diameter). The yellow arrows indicate the implantation of scaffolds.

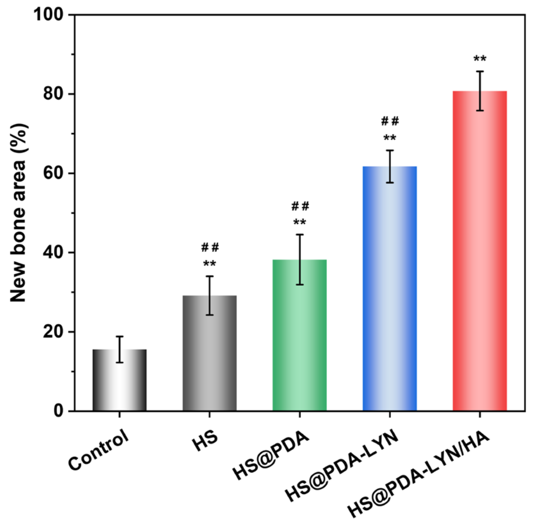

Supplementary Figure 12. Quantitative analysis of new bone area in different groups at 8 weeks. Data are expressed as the mean ± SD (n = 4). *P < 0.05 and **P < 0.01 indicate significant differences compared with the control group. #P < 0.05 and # #P < 0.01 indicate significant differences compared with the HS@PDA-LYN/HA group.

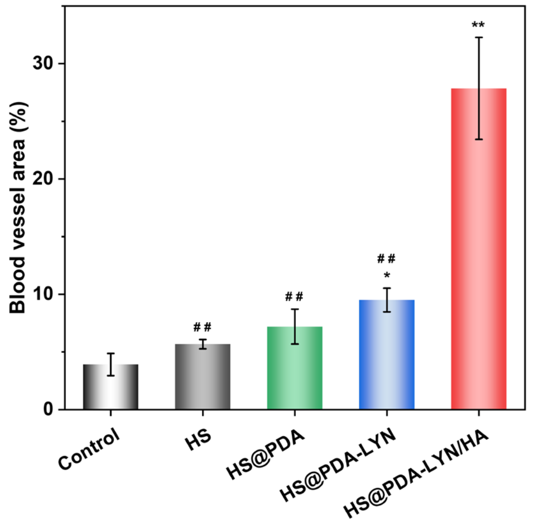

Supplementary Figure 13. Quantitative analysis of blood vessel area in different groups at 8 weeks. Data are expressed as the mean ± SD (n = 4). *P < 0.05 and **P < 0.01 indicate significant differences compared with the control group. #P < 0.05 and # #P < 0.01 indicate significant differences compared with the HS@PDA-LYN/HA group.

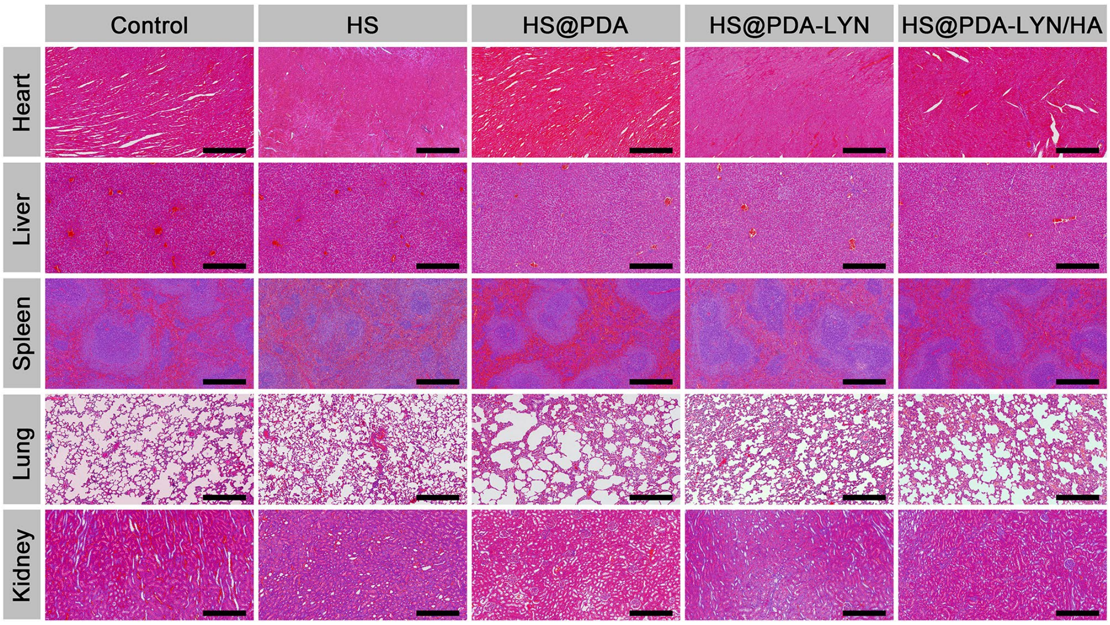

Supplementary Figure 14. H&E staining of the major organs, including the heart, liver, spleen, lung, and kidney, harvested from different groups. Scale bar: 200 μm.

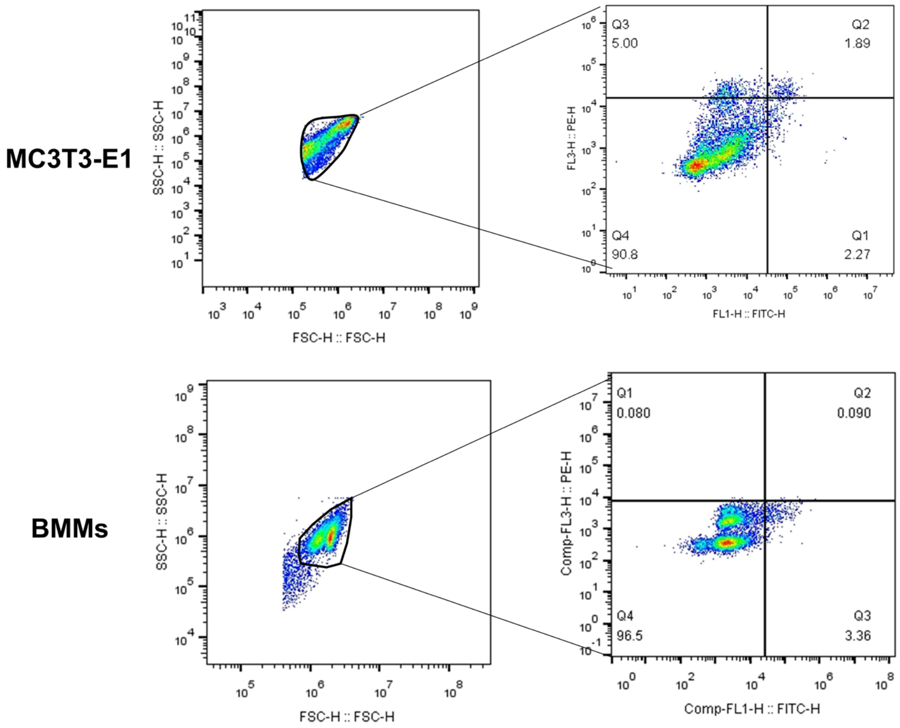

Supplementary Figure 15. Sorting Strategy for the Annexin V FITC assay relate to **Figure 3c**. Cells were first gated on FSC/SSC to eliminate fragments and then assayed for apoptosis based on cell positivity with Annexin V FITC/Propidium Iodide.

Supplementary Table 1. Primers and sequences used in this study.

Gene	Primers Sequence (F, forward; R, reverse)	
Runx2	F	5’-AATCCACAAGGACAGAGTCAGAT-3′
	R	5’-ACTGCCTGGGGTCTGAAAAAG-3′
Col-1	F	5’-ACGCCATCAAGGTCTACTGC-3′
	R	5’-ACTCGAACGGGAATCCATCG-3′
OPN	F	5’-CATTCTCGGAGGAAACCAGC-3′
	R	5’-GAATTCAGCCAGGAGAACTGC-3′
NFATc1	F	5’-TATATGAGCCCATCCTTGCCT-3′
	R	5’-GGCTGCCTTCCGTCTCATAG-3′
CTSK	F	5’-GCACCCTTAGTCTTCCGCTC-3′
	R	5’-GGTCATATAGCCGCCTCCAC-3′
RANK	F	5’-CTCCTTGGAAAGCTAGAAGCAC-3′
	R	5’-TTCCCTCCCTTCCTGTAGTAAAC-3′
ULK1	F	5’-CCCATCCTAGGCTCTCCTACC-3′
	R	5’-AGAGGCCTGAGTCCCAAATG-3′
GAPDH	F	5’-TGAAGGGTGGAGCCAAAAG-3′
	R	5’-AGTCTTCTGGGTGGCAGTGAT-3′

Abbreviations: Runx2, runt-related transcription factor 2; Col-1, type I collagen; OPN, osteopontin; NFATc1, nuclear factor of activated T cells 1; CTSK, cathepsin K; RANK, receptor activator of NF-κB; ULK1, UNC-51-like kinase 1.
